# Transcriptional regulation of monolignol *O*‐methylation in *Cleome hassleriana*

**DOI:** 10.1111/tpj.71123

**Published:** 2026-09-21

**Authors:** Chunliu Zhuo, David Burks, Xirong Xiao, Rajeev K. Azad, Xiaolan Rao, Fang Chen, Richard A. Dixon

**Affiliations:** ^1^ BioDiscovery Institute and Department of Biological Sciences University of North Texas Denton Texas 76203 USA; ^2^ Center for Bioenergy Innovation (CBI), Oak Ridge National Laboratory Oak Ridge Tennessee 37831 USA; ^3^ Department of Plant and Soil Sciences Oklahoma State University Stillwater Oklahoma 74078 USA; ^4^ Ambry Genetics, One Enterprise Aliso Viejo California 92656 USA; ^5^ State Key Laboratory of Biocatalysis and Enzyme Engineering, School of Life Sciences Hubei University Wuhan 430062 China; ^6^ Center for Biotechnology and Genomics Texas Tech University Lubbock Texas 79409 USA

**Keywords:** *Cleome hassleriana*, C‐lignin, hairy root, monolignol biosynthesis, MYB transcription factor, seed coat

## Abstract

Catechyl (C) lignin, a linear homopolymer restricted to seed coats of a limited number of plant species, has favorable properties for chemical and biomaterial manufacture. In the seed coat of *Cleome* *hassleriana*, lignin composition switches from guaiacyl (G‐) lignin to C‐lignin during development. This involves loss of expression of caffeoyl‐CoA 3‐*O*‐methyltransferase (ChCCoAOMT) and caffeic acid 3‐*O*‐methyltransferase (ChCOMT), and upregulation of a cinnamyl alcohol dehydrogenase (ChCAD5) and laccase (ChLAC8) with substrate preference for the caffeyl moiety. How the loss of monolignol *O*‐methylation is regulated is not understood. We reconstructed coexpression networks of the above enzymes with transcription factor (TF) candidates based on RNA sequencing data. Using a dual‐luciferase reporter assay, we identified ChMYB20 as an activator of the ChCCoAOMT and ChCOMT promoters, and ChMYB6‐like as a repressor. ChMYB5 and ChMYB42 induced ChCAD5, ChLAC8, and ChMYB6‐like, and ChNAC92‐like induced ChMYB5 and ChMYB42. The roles of the TFs in the network were further confirmed by expressing them in *Cleome* hairy roots. ChMYB5 and ChMYB42 suppressed monolignol *O*‐methyltransferase genes, activated downstream C‐lignin‐associated genes, and also activated each other, suggesting they are players in the switch from G‐ to C‐lignin biosynthesis via blockage of monolignol *O*‐methylation. Models for the gene regulatory networks are presented.

## Significance Statement

Catechyl (C) lignin, a linear homopolymer restricted to seed coats of a limited number of plant species, has favorable properties for chemical and biomaterials manufacture. Here, we utilize transcript coexpression and promoter activity assays to uncover features of the transcriptional control of C‐lignin in the seed coat of *Cleome hasleriana.*

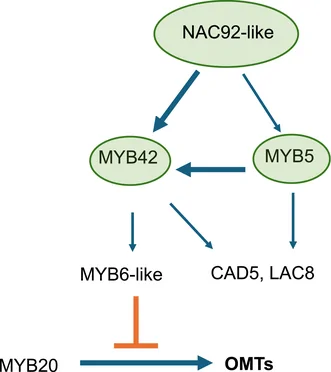

C‐lignin has favorable properties for chemical and biomaterial manufacture. Its biosynthesis requires the suppression of the canonical monolignol *O*‐methylation reactions. We have identified transcription factors expressed in the seed coat of *Cleome hassleriana* that are associated with the onset of C‐lignin biosynthesis through their suppression of monolignol *O*‐methylation and activation of downstream reactions in the C‐lignin pathway.

## INTRODUCTION

C‐Lignin, first discovered in the seed coat of the orchid *Vanilla planifolia* (Chen et al., 2012), is a homopolymer of the monolignol caffeyl alcohol, linked as unbranched chains through benzodioxane units. Its unique chemical and physical properties have made it a target for lignin valorization efforts to provide a value‐added co‐product for biomass/bioenergy crops such as poplar and switchgrass (Dixon et al., 2024; Li et al., 2018; Ragauskas et al., 2014; Stone et al., 2018). Although several reports document the presence of C‐lignin in seed coats of mainly non‐crop plants (Barsberg et al., 2018; Chen et al., 2013; Tobimatsu et al., 2013; Wang et al., 2021), to the best of our knowledge there are currently no reports of C‐lignin as a natural component of vegetative biomass in any plant species.

The ornamental plant *Cleome hassleriana* has been developed as a model system for studying the biosynthesis of C‐lignin. This species produces classical guaiacyl (G)/syringyl (S) lignin in its stems and G‐lignin in its seed coat for the first 12 days after pollination (DAP), thereafter producing only C‐lignin in the seed coat (Tobimatsu et al., 2013; Zhuo et al., 2022). *C. hassleriana* (*Cleome*) has a sequenced genome (Cheng et al., 2013), and is genetically transformable (Wang et al., 2020). We have previously generated a transcriptome database for *Cleome* seed coats after various stages of development, covering the period of the switch from G‐ to C‐lignin production (Zhuo et al., 2019). Interrogation of these data, coupled to biochemical analyses, has shown that the onset of C‐lignin biosynthesis is associated with the transcriptional downregulation of genes encoding caffeoyl‐CoA 3‐*O*‐methyltransferase (CCoAOMT) and caffeic acid 3‐*O*‐methyltransferase (COMT), the two enzymes responsible for monolignol *O*‐methylation (Zhuo et al., 2019). C‐lignin formation in *Cleome* seed coat is also associated with upregulation of a form of cinnamyl alcohol dehydrogenase (ChCAD5) that exhibits a preference, but not absolute specificity, for caffealdehyde over coniferaldehyde, and of a laccase (ChLAC8) that oxidizes caffeyl alcohol preferentially to coniferyl alcohol (Wang et al., 2020; Zhuo et al., 2019). Metabolic flux analysis has indicated that different routes to the monolignols may be utilized during the periods of G‐ and C‐lignin biosynthesis (Zhuo et al., 2022).

Based on a phylogenetic analysis of C‐lignin occurrence among the Cactaceae (Chen et al., 2013), it is likely that the ability to make C‐lignin has evolved recently and often, suggesting that few new steps are involved. The simplest model to account for the evolution of C‐lignin biosynthesis and the rapid switch from G‐ to C‐lignin synthesis in *Cleome* is an alteration in the transcriptional control of a set of genes that also functions in classical lignin biosynthesis, such as the *COMT* and *CCoAOMT* genes (Ha et al., 2023).

Gene regulatory networks (GRNs) have been described for lignin biosynthesis in several systems, including during wood formation in poplar (Lin et al., 2013) and inducible lignin formation in switchgrass suspension cells (Rao et al., 2019). In these cases, several layers of TFs operate between a master regulatory gene and MYB family TFs that recognize multiple monolignol biosynthetic gene promoters. We here report the identification of TFs that regulate the expression of *ChCCoAOMT*, *ChCOMT*, and downstream monolignol biosynthesis genes expressed in the Cleome seed coat. The switch from G‐ to C‐lignin biosynthesis is associated with the downregulation of ChMYB20, a TF that transcriptionally activates both *ChCOMT* and *ChCCoAOMT* genes, and the upregulation of ChMYB6‐like, a transcriptional repressor that potently blocks transcription induced by ChMYB20. Additional members of the regulatory network were identified which regulate the expression of ChCAD5, ChLAC8, and ChMYB6‐like, and the model was subjected to preliminary testing by TF overexpression in Cleome hairy roots. However, although altering the expression of these TFs alters OMT expression and lignin composition in Cleome hairy roots, it was still not sufficient to allow for accumulation of significant levels of C‐lignin in this tissue.

## RESULTS

### Construction of a WGCNA network to define genes associated with C‐lignin biosynthesis in *cleome*

To identify genes specifically associated with C‐lignin biosynthesis, we built an unsigned network using the Weighted Gene Coexpression Network Analysis (WGCNA) platform (Langfelder & Horvath, 2008) with the RNA‐Seq datasets sourced from the seed coats of *Cleome hassleriana* (Zhuo et al., 2019). Unsigned networks allow for the identification of both activators and repressors. Samples were harvested at 2‐day intervals throughout development from 8 to 18 days after pollination (DAP) with three replicates. A sample‐to‐sample Pearson correlation map was constructed to overview the datasets (Figure S1). We observed three strong correlations (more than 75%) of transcript profiles at 8 and 10 DAP, 10–16 DAP, and 12–18 DAP (Figure S1). G‐lignin accumulates in the seed coats for the first 6–12 DAP, after which C‐lignin is deposited (Tobimatsu et al., 2013). These results suggest that changes in gene expression associated with C‐lignin biosynthesis more likely occurred during 10–12 DAP.

A Salmon pseudoalignment was generated using the normalized transcript profiles. All 18 datasets contained more than 60% reads aligning to the published *C. hassleriana* (Cleome) transcriptome (ASM46358v1, Cheng et al., 2013) with 15–22% of transcripts represented, which is a result of the seed coats being the only tissue source while the reference transcriptome was generated from leaf tissue (Figure S2). After filtering, a cluster dendrogram was generated to show the hierarchical relationship between datasets (Figure S3). Among time points, transcript profiles at 14 and 16 DAP were the most similar, the next two most similar time points being 10 and 12 DAP. Datasets of 10–16 DAP were more similar to each other than they were to datasets at 8 or 18 DAP. A total of 28 modules were found in the WGCNA‐derived networks. Module sizes ranged from 12 to 26 331 transcripts.

### Modules associated with lignin biosynthesis in *cleome* seed coats

Genes associated with the Cleome monolignol pathway were found in six of the 28 modules of the network (Table 1). ChCCoAOMTs, ChCOMTs, and ChCAD4, which exhibit a preference for coniferaldehyde over caffealdehyde, were found in Module 1, suggesting that Module 1 is associated with G‐lignin biosynthesis. ChCAD5, which exhibits a preference for caffealdehyde over coniferaldehyde, along with other monolignol pathway genes, was found in Module 2, suggesting that Module 2 is the main player during C‐lignin biosynthesis. Interestingly, the highly expressed cinnamoyl‐CoA reductase ChCCR1 was not found in Modules 1 or 2. ChLAC8, which exhibits a preference for caffeyl alcohol over coniferyl alcohol, along with ChCCR1, was found in Module 4. ChC3H, Ch4CL4, and ChC4H2 did not group with any monolignol genes. They were found in Modules 3, 5, and 25, respectively. To address the transcriptional regulation of C‐lignin biosynthesis, we examined the transcription factors (TFs) in Modules 1, 2, and 4.

**Table 1:** Module membership for *Cleome* monolignol pathway genes

Module	Transcript ID	Annotation	8DAP	10DAP	12DAP	14DAP	16DAP	18DAP
1	XM_010555029.2	PAL3, L‐phenylalanine ammonia‐lyase 1‐like	182	255	415	522	408	150
1	XM_010549701.1	**CCoAOMT1**, caffeoyl‐CoA *O*‐methyltransferase 1	1137	769	150	72	103	117
1	XM_010528660.1	**CCoAOMT5**, caffeoyl‐CoA *O*‐methyltransferase 5	702	599	186	75	50	31
1	XM_010538744.2	**COMT1**, flavone 3’‐*O*‐methyltransferase 1	570	424	54	12	8	12
1	XM_010522631.1	**COMT2**, flavone 3’‐*O*‐methyltransferase 1‐like	277	111	15	11	8	2
1	XM_010520990.1	CAD4, cinnamyl alcohol dehydrogenase 4	167	159	93	89	72	33
1	XM_010532430.2	CAD7, cinnamyl alcohol dehydrogenase 5‐like	91	82	31	17	14	8
1	XM_010533126.2	CAD8, probable cinnamyl alcohol dehydrogenase 9	201	129	95	72	57	30
2	XM_010529772.2	PAL1, L‐phenylalanine ammonia‐lyase 1	335	392	409	400	392	204
2	XM_010531425.2	PAL2, L‐phenylalanine ammonia‐lyase 1‐like	55	105	102	91	93	28
2	XM_010545242.2	PAL4, L‐phenylalanine ammonia‐lyase 4	228	312	255	333	278	118
2	XM_010523713.2	C4H1, *trans*‐cinnamate 4‐monooxygenase‐like	633	716	805	957	782	426
2	XM_010543479.2	4CL1, 4‐coumarate‐CoA ligase 1‐like	619	802	682	1096	1031	359
2	XM_010528803.1	HCT, shikimate *O*‐hydroxycinnamoyltransferase	669	814	778	987	804	278
2	XM_010536823.1	CSE, caffeoylshikimate esterase	195	246	265	350	297	130
2	XM_019201334.1	**CAD5**, cinnamyl alcohol dehydrogenase 5‐like	128	409	595	661	496	180
3	XM_010527130.2	C3H, cytochrome P450 98A3	403	405	425	407	237	84
4	XM_010539570.1	CCR1, cinnamoyl‐CoA reductase 1	541	844	1583	2955	2783	1135
4	XM_010555066.2	**LAC8**, laccase 8	0	7	66	141	138	40
5	XM_010547317.2	4CL4, 4‐coumarate‐CoA ligase 4	109	173	93	88	51	15
25	XM_010544397.2	C4H2, *trans*‐cinnamate 4‐monooxygenase	149	163	400	438	295	108

Genes associated with the monolignol pathway were found in six of the 28 modules of the unsigned network built with the transcriptome dataset for *Cleome* seed coats (Zhuo et al., 2019) using the WGCNA platform. Transcript levels of genes are means of three biological replicates. Units are FPKM (fragments per kilobase of transcript per million mapped reads). DAP, days after pollination. Intensity of  green shading reflects  transcript level. Genes in bold are modulated during C‐lignin biosynthesis.

### ChMYB20 and ChMYB6‐like TFs target genes for monolignol 3‐*O*‐methylation

**Figure 1:**
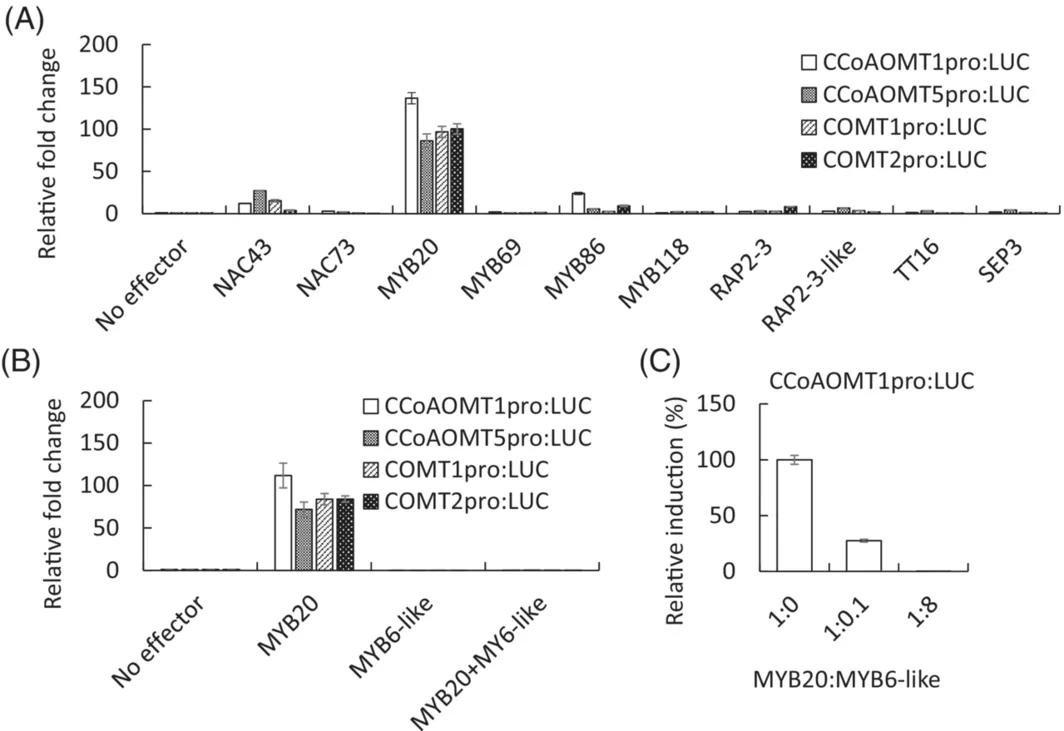
Promoter transactivation by ChMYB20 and ChMYB6‐like. (A) ChMYB20 activates the 3‐*O*‐methyltransferase gene promoters in transient promoter transactivation assays. Promoters of CCoAOMTs 1 and 5, and COMTs 1 and 2, were cloned and linked to the reporter firefly luciferase. The TFs were cloned and expressed under the control of the 35S promoter in the effector vector. Promoter, reporter and internal control plasmids were co‐transfected into Arabidopsis protoplasts. Dual‐luciferase reporter assays were performed after 20 h of incubation. Data are means of duplicate measurements of three biological replicates. (B) ChMYB6‐like represses the activation of 3‐*O*‐methyltransferase gene promoters by ChMYB20. Experiments were carried out as in (A) but also testing the effect of ChMYB6 alone or in concert with MYB20. (C) Dosage‐dependent suppression of ChMYB20 activity by ChMYB6‐like in transient expression assays using Arabidopsis protoplasts. Data are means of duplicate measurements of three biological replicates.

Previously, we showed that the switch to C‐lignin formation in the Cleome seed coat was accompanied by downregulation of transcripts encoding ChCCoAOMT and ChCOMT, each of which is encoded by two genes that are expressed in the seed coat (Zhuo et al., 2019). Activator candidates in Module 1 are expected to share similar expression patterns with ChCOMTs and ChCCoAOMTs (expressed early, downregulated around 12 DAP), whereas TFs with elevated transcript levels from around 12 DAP are potential repressor candidates. To target TF candidates, we sub‐clustered modules using the Markov Cluster Algorithm with an inflation factor of 1.8. Based on this analysis, 10 activator candidates were identified (Table 2a). To determine if any of these candidates could activate the four ChCOMT and ChCCoAOMT promoters, we conducted dual‐luciferase reporter assays for promoter transactivation using transient expression in *Arabidopsis thaliana* protoplasts. The ChCOMT and ChCCoAOMT promoters were first cloned in front of the firefly luciferase reporter gene. Test TFs were expressed under the control of the 35S promoter in the effector vector. The reporter constructs were then co‐transfected into the protoplasts with the various effectors. Among the activator candidates, ChMYB20 activated all four ChCOMT and ChCCoAOMT promoters strongly, by 86‐ to 136‐fold, and ChNAC43 and ChMYB86 activated the promoters by 3‐ to 27‐fold (Figure 1A). These results suggest that ChMYB20 is a major activator of ChCOMT and ChCCoAOMT transcription in the seed coat of Cleome, and that the two pairs of *COMT* and *CCoOAMT* genes respond similarly.

**Table 2:** Transcript levels of transcription factor candidates for association with C‐lignin biosynthesis

(a) Transcription factor candidates for OMTs.								
Module	Transcript ID	Annotation	8DAP	10DAP	12DAP	14DAP	16DAP	18DAP
		**NAC domain protein**						
1	XM_010546573.2	NAC43, NAC domain‐containing protein 43	56	50	4	0	0	0
1	XM_010528198.1	NAC73, NAC domain‐containing protein 73	91	60	3	0	0	0
		**MYB domain protein**						
1	XM_010531859.2	MYB6‐like, transcription repressor	6	11	20	24	26	28
1	XM_010553457.1	MYB20, protein ODORANT1‐like	57	51	3	0	0	0
1	XM_010549589.2	MYB69, transcriptional activator MYB‐like	15	10	7	4	4	2
1	XM_010550874.2	MYB86, transcription factor	22	24	7	2	1	0
1	XM_019201546.1	MYB118, uncharacterized	84	57	22	6	1	0
		**AP2 domain protein**						
1	XM_010526253.2	RAP2‐3, ethylene‐responsive transcription factor RAP2‐3	378	364	221	149	118	90
1	XM_010550674.2	RAP2‐3‐like, ethylene‐responsive transcription factor	795	551	260	123	73	83
		**MADS‐box protein**						
1	XM_010523748.1	TT16, TRANSPARENT TESTA 16	107	71	32	9	4	2
1	XM_010532259.2	SEP3, developmental protein SEPALLATA 3‐like	27	22	20	9	9	8

(b) Transcription factor candidates for CAD5 and LAC8.								
Module	Transcript ID	Annotation	8DAP	10DAP	12DAP	14DAP	16DAP	18DAP
		**WRKY domain TF**						
25	XM_010553096.1	WRKY57, probable WRKY transcription factor 57	24	45	76	76	65	37
		**NAC domain TF**						
25	XM_010559131.1	NAC40, NAC domain‐containing protein 40	5	7	12	10	7	4
1	XM_010560406.2	NAC92‐like, NAC domain‐containing protein 92‐like	6	22	23	18	17	12
		**MYB domain TF**						
2	XM_010544164.2	MYB5, transcription repressor MYB5	13	14	15	20	13	5
1	XM_010541557.2	MYB42, protein ODORANT1‐like	18	30	140	224	156	71
		**AP2 domain TF**						
2	XM_010527524.2	ERF015, ethylene‐responsive transcription factor	41	128	171	199	172	83
4	XM_010533924.1	ERF105, ethylene‐responsive transcription factor	21	21	37	130	127	57
2	XM_010543298.2	SHINE2, ethylene‐responsive transcription factor	29	62	83	98	81	29
		**MADS‐box TF**						
4	XM_010527034.2	AGL1, agamous‐like MADS‐box protein	215	254	311	396	319	136
4	XM_010544569.2	AGL11, agamous‐like MADS‐box protein	63	61	149	304	417	219
		**bZIP domain TF**						
25	XM_010547928.2	bZIP43, basic leucine zipper	78	261	518	594	422	142
1	XM_010541582.2	bZIP44, bZIP transcription factor	164	245	276	389	322	161
4	XM_010534319.2	SUP, transcriptional regulator SUPERMAN	3	4	12	14	19	7
4	XM_010537944.2	ZAT12, zinc finger protein	2	3	4	9	10	6

The list of transcription factor candidates for OMTs (a), and CAD5 and LAC8 (b) were generated based on the WGCNA network and Pearson correlation. To obtain an appropriate size of the TF candidate pool, an inflation factor of 1.8 was used to subcluster Module 1 with the Markov Cluster Algorithm and target 11 TF candidates for OMTs. There were only a few TFs in Modules 2 and 4; additional candidates that strongly correlated with CAD5 or LAC8 were therefore included (correlation coefficient >0.8). Transcript levels of genes are means of three biological replicates. Units are FPKM (fragments per kilobase of transcript per million mapped reads). DAP, days after pollination. Darker green shading indicates higher transcript levels of the candidate transcription factor.

MYB6‐like was the only gene identified as a repressor candidate (negative correlation in Table 2a). To test whether ChMYB6‐like can suppress the activation of ChCOMT and ChCCoAOMT promoters, we co‐transfected ChMYB6‐like into Arabidopsis protoplasts along with an equal amount of plasmid harboring the activator ChMYB20. ChMYB6‐like could completely suppress the activation of ChCOMT and ChCCoAOMT promoters by ChMYB20 (Figure 1B). To better define the interference of ChMYB6‐like on ChMYB20, we tested the dosage‐dependent suppression of ChCCoAOMT1 promoter activity. In Cleome seed coats, ChMYB20 is expressed at the G‐lignin stage, whereas ChMYB6‐like is expressed later during seed development when C‐lignin is produced (Table 2a). We therefore combined ChMYB20 and ChMYB6‐like in the co‐transfection experiments according to their transcript level ratio during seed development. When the ratio of ChMYB20 to MYB6‐like plasmids was 1:0.1, as their corresponding transcripts are at the G‐lignin stage, ChMYB6‐like caused a 72% reduction of ChCCoAOMT1 promoter activity (Figure 1C). When the ratio was 1:8, as it is at the later stage of seed development, ChMYB6‐like completely suppressed *ChCCoAOMT1* promoter activity (Figure 1C). These results suggest that the balance between ChMYB20 and ChMYB6‐like may regulate OMT expression in the seed coat of Cleome, favoring C‐lignin biosynthesis as the ratio of repressor to activator increases.

### TFs modulating expression of genes positively associated with C‐lignin biosynthesis

**Figure 2:**
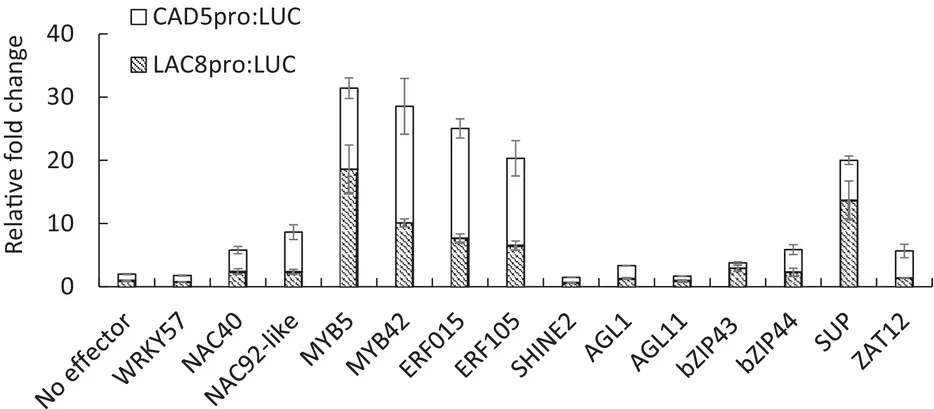
*In vitro* transactivation of the ChCAD5 and ChLAC8 promoters. ChMYB5 and ChMBY42 activate the promoters of ChCAD5 and ChLAC8 in transient expression assay using Arabidopsis protoplasts. Promoters of ChCAD5 and ChLAC8 were cloned and linked to the reporter firefly luciferase. The test transcription factors were cloned and constructed under the control of the 35S promoter in the effector vector. Together with the internal control plasmids, they were transfected into the Arabidopsis protoplasts. Dual‐luciferase reporter assay was performed after 20 h of incubation. Data are means of duplicate measurements of three biological replicates.

ChCAD5 is the cinnamyl alcohol dehydrogenase that is most highly expressed in the Cleome seed coat at the stage of C‐lignin biosynthesis, and it shows a preference for caffealdehyde over coniferaldehyde. Downregulation of ChCAD5 reduces C‐lignin accumulation in the Cleome seed coat (Zhuo et al., 2019). Its expression is low but measurable as early as 8 DAP and then increases and remains high through 16 DAP (Table 1) (Zhuo et al., 2019), explaining why it is found in the same WGCNA module (Module 2) as many of the genes common to both G and C‐lignin biosynthesis (Table 1). ChLAC8, associated with C‐lignin polymerization (Wang et al., 2020), was present in WGCNA Module 4. Because there were only a few TFs in Modules 2 and 4, we included additional TFs with a correlation coefficient greater than 0.80. These additional candidates belonged to Modules 1 and 25. Fourteen TF candidates were identified (Table 2b). These TFs are expressed maximally at 12–16 DAP. Their open reading frames were cloned and tested as effectors for activation of the ChCAD5 and ChLAC8 promoters in Arabidopsis protoplasts as above. Of these, ChMYB5, ChMYB42, ChERF015, ChERF105, and ChSUP were able to transactivate both promoters (Figure 2). Because of the general involvement of MYB TFs in lignin GRNs (Lin et al., 2013; Rao et al., 2019; Zhong et al., 2007), and to simplify analyses, we concentrated further studies on ChMYB5 and ChMYB42.

### Higher order regulators of MYB TFs associated with C‐lignin biosynthesis

**Figure 3:**
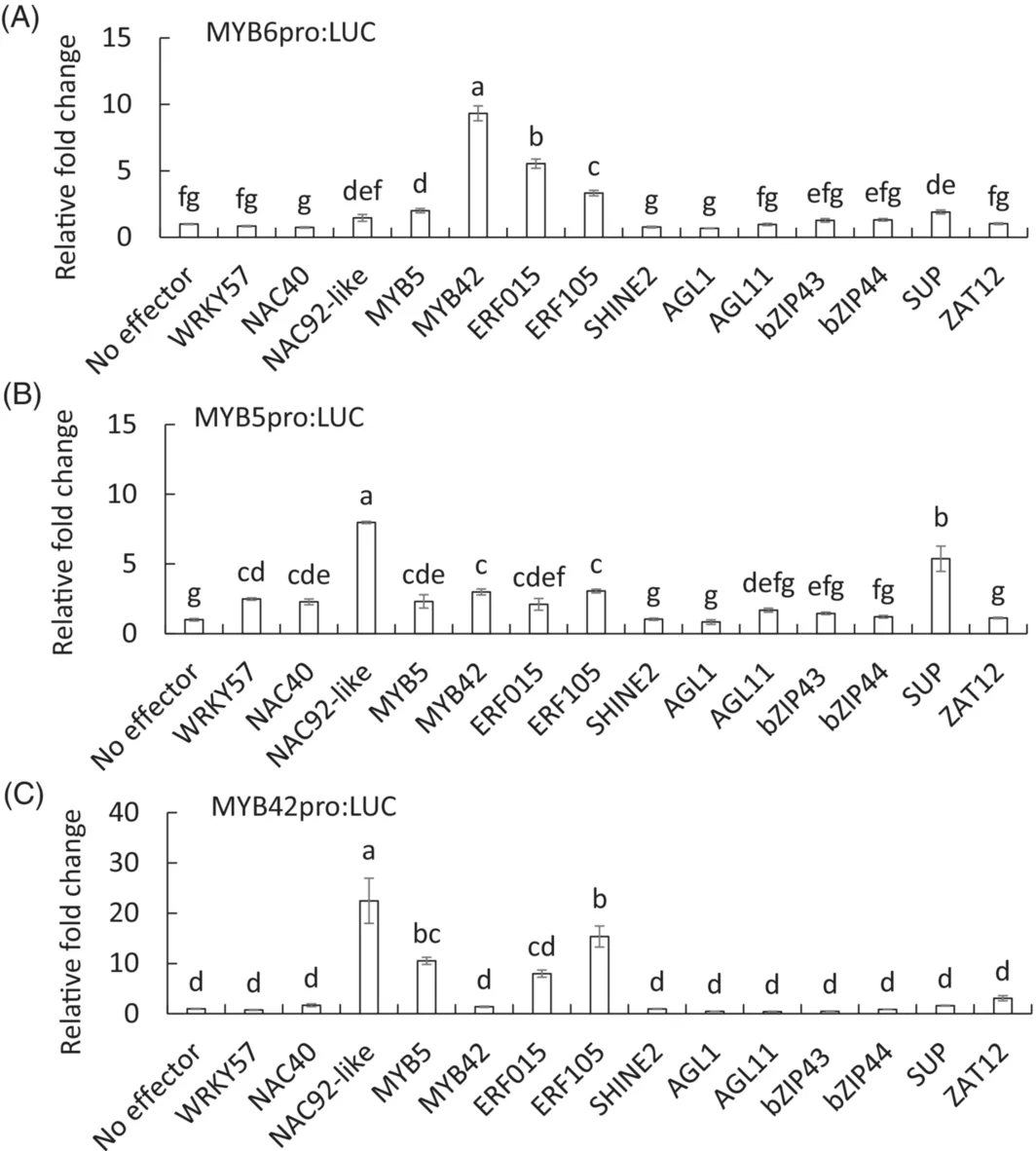
*In vitro* transactivation of the ChMYB6‐like, ChMYB5 and ChMYB42 promoters. (A) ChMYB42 and ChERF105 activate the promoter of ChMYB6‐like in transient expression assay using Arabidopsis protoplasts. (B, C) ChNAC92‐like activates the promoters of ChMYB5 and ChMYB42. Promoters were cloned and linked to the reporter firefly luciferase. The test transcription factors were cloned and constructed under the control of the 35S promoter in the effector vector. Together with the internal control plasmids, they were transfected to the Arabidopsis protoplasts. Dual‐luciferase reporter assay was performed after 20 h of incubation. Data are means of duplicate measurements of three biological replicates. Letters indicate significant differences at the *P* ≤ 0.05 level by one‐way ANOVA (Duncan) with SPSS Statistics.

In all models of lignin GRNs, higher order TFs act to regulate downstream MYB TFs (Chen et al., 2019; Lin et al., 2013; Rao et al., 2019; Zhao, 2016). We therefore selected the TFs associated with C‐lignin (Table 2b), to test their effects on transactivation of the ChMYB6‐like, ChMYB5 and ChMYB42 promoters in the Arabidopsis protoplast assay. The results revealed ChMYB42 and ChERF015 as weak activators of ChMYB6‐like transcription, ChNAC92‐like and ChSUP as weak activators of ChMYB5, and ChNAC92‐like and ChERF105 as moderately strong activators of ChMYB42 (Figure 3).

MYB5 has been shown to act synergistically with other TFs to activate pathways of flavonoid biosynthesis (Shen et al., 2019; Xu et al., 2015), including the TTG1/TT8 complex associated with proanthocyanidin biosynthesis in the Arabidopsis seed coat (Baudry et al., 2004; Liu et al., 2014). To determine whether ChMYB5 acted with partners to activate the ChMYB42 promoter, we tested the effects of co‐transfection of ChMYB5 with ChNAC92‐like or AtTTG1/TT8 on expression of the ChMYB42 promoter in Arabidopsis protoplasts. No strong synergistic effects were observed, although addition of AtTTG1/TT8 increased the activation of the ChMYB42 promoter by ChMYB5 (Figure S4).

We next performed a phylogenetic analysis of ChMYB20, ChMYB6‐like, ChMYB5, and ChMYB42 together with other MYBs known to regulate secondary cell wall biosynthesis or phenylpropanoid metabolism (Figure S5). In general, these MYBs formed three clades: activators (blue), repressors (orange), and a third clade (purple). ChMYB20 and ChMYB42 are closely related to AtMYB20, AtMYB43, AtMYB42, AtMYB85, and PtMYB1 that activate lignin biosynthesis during secondary cell wall formation (Geng et al., 2020; Patzlaff et al., 2003). ChMYB6‐like is closely related to AtMYB6, AtMYB3, ZmMYB8, and AmMYB330 that repress the phenylpropanoid pathway (Fornalé et al., 2006; Ma & Constabel, 2019; Tamagnone et al., 1998; Zhou et al., 2017). Lastly, ChMYB5 is closely related to AtMYB75, VvMYB5a, and PtrMYB6 that regulate distribution among phenylpropanoid‐derived compounds (Bhargava et al., 2010; Deluc et al., 2006; Wang et al., 2019). These MYBs belong to the 2R group containing the R2 and R3 DNA binding domains at their N‐terminal regions (Figure S6). ChMYB20 and ChMYB42 do not have any motifs at their C‐termini (Figure S6a) and belong to Subgroup 28 (Liu et al., 2015). ChMYB6‐like contains two typical motifs of Subgroup 4 at its C‐terminal (Figure S6b): C1 (LIsrGIDPxT/SHRxI/L) and C2 (pdLNL[D/E]Lxi[G/S]) (Liu et al., 2015; Stracke et al., 2001). The latter is also known as a type of ethylene‐responsive element binding factor‐associated amphiphilic repression (EAR) motif (LxLxL or DLNx(x)P) (Kagale et al., 2010). ChMYB5 belongs to subgroup 27, which contains C1 and C3 (DDxF[S/P]SFL[N/D]SLIN[E/D]) motifs and the bHLH binding domain ([D/E]Lx2[R/K]x3Lx6Lx3R) (Zimmermann et al., 2004) (Figure S6c). The promoters of ChCOMTs, ChCCoAOMTs, ChCAD5, and ChLAC8 contain several putative MYB‐binding sites, including AC elements with the consensus sequences ACCTNNC (Mellway et al., 2009) and secondary wall MYB‐responsive elements (SMRE) with the consensus sequences ACC(A/T)A(A/C)(T/C) (Zhong & Ye, 2012) (Figure S7).

### Reconstructing gene regulatory networks for monolignol *O*‐methylation in cleome hairy roots; the MYB6‐like/MYB20 module

Testing potential GRNs in the Cleome seed coat is time consuming because of the need for stable transformation and growing of T_2_ plants to the seed stage. We therefore decided to see if we could reconstruct the GRNs in Cleome hairy roots. Transgenic hairy roots can be rapidly generated and have been demonstrated previously to support low‐level C‐lignin accumulation in *Medicago truncatula* (Ha et al., 2023). However, it must be cautioned that it is unclear whether ectopic expression of TFs in hairy roots directly mimics their involvement in transcriptional control in the seed coat.

**Figure 4:**
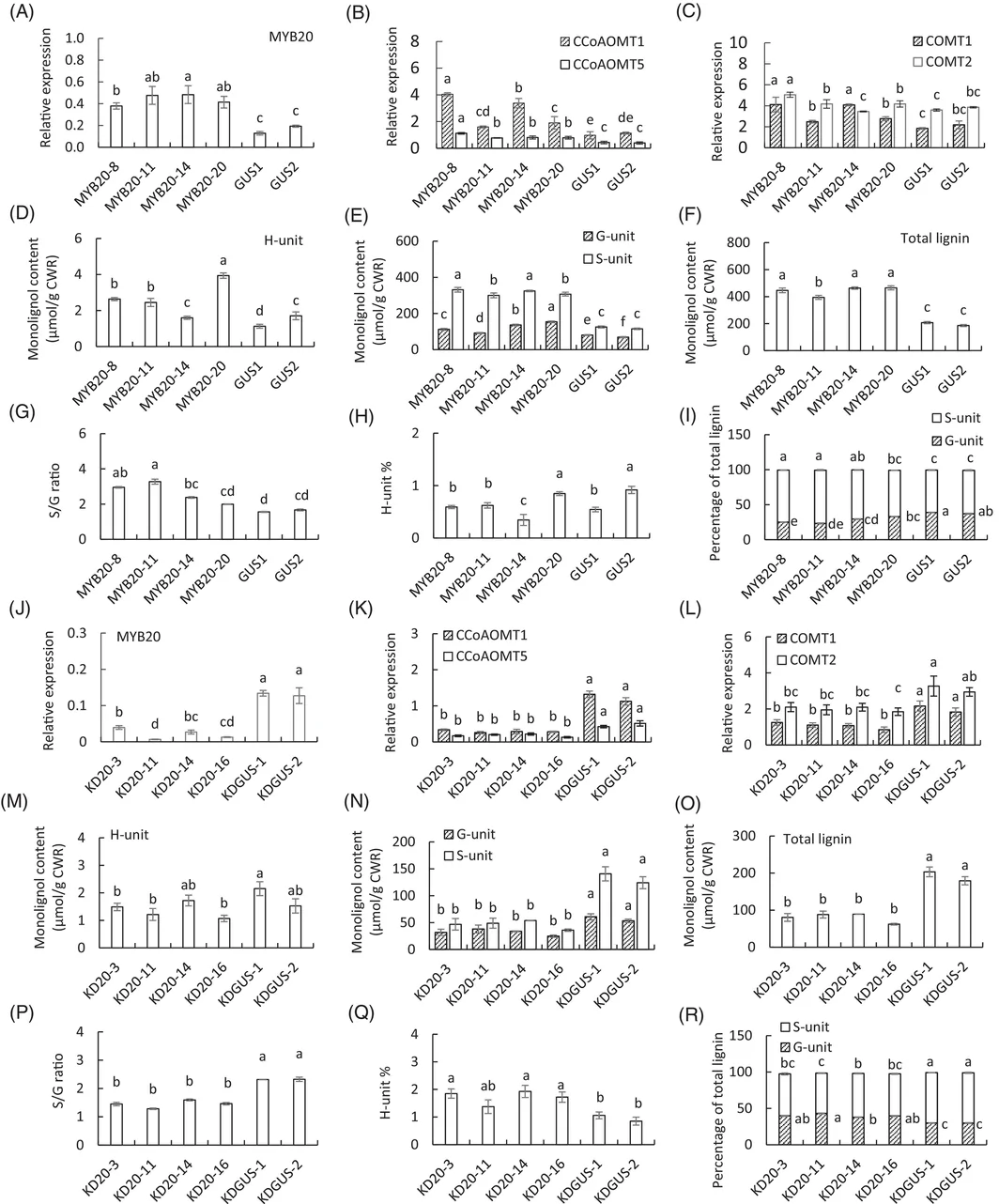
ChMYB20 regulates lignin content and composition in Cleome hairy roots. (A–C, J–L) Transcript levels of ChMYB20 (A, J), ChCCoAOMT1 and ChCCoAOMT5 (B, K), and ChCOMT1 and ChCOMT2 (C, L) as determined by qRT‐PCR, expressed relative to ubiquitin‐conjugating enzyme E2 11 (UBC), in ChMYB20‐overexpressing (A–C) or ChMYB20 knockdown (J–L) hairy roots. (D–F, M–O) Lignin monomer thioacidolysis yields for H (D, M), G and S (E, N) and total (F, O) units in ChMYB20‐overexpressing (D–F) or ChMYB20 knockdown (M–O) hairy roots. CWR, cell wall residue. (G, P) S/G ratios as determined by thioacidolysis in ChMYB20‐overexpressing (G) or ChMYB20 knockdown (P) hairy roots. (H, I, Q, R) Lignin monomer thioacidolysis yields for H (H, Q) and G and S (I, R) units as percentages of total lignin units in ChMYB20‐overexpressing (H, I) or ChMYB20 knockdown (Q, R) hairy roots. Four independent lines overexpressing ChMYB20 or expressing an RNAi construct for knocking down expression of ChMYB20 were analyzed and compared with two lines expressing GUS. Data are means of three biological replicates. Letters indicate significant differences at the *P* ≤ 0.05 level by one‐way ANOVA (Duncan) with SPSS Statistics.

In the first set of experiments, we expressed ChMYB20 under the control of the cauliflower mosaic virus 35S promoter. Twenty and five independent transgenic Cleome hairy root lines expressing ChMYB20 or a β‐glucuronidase (*GUS*) gene as control were generated, respectively (Figure S8a). Based on the transcript levels of ChMYB20, we selected four independent lines overexpressing ChMYB20 and two GUS‐expressing control lines for further study (Figure 4A). Overexpression of ChMYB20 significantly induced transcript levels of ChCCoAOMTs 1 and 5, by 1.5‐ to 3.8‐fold compared with the GUS control, respectively, resulting in a log_2_ fold change of 0.58 to 1.93 (Figure 4B). Transcript levels of ChCOMTs 1 and 2 were also weakly induced in two of the ChMYB20‐overexpressing lines (Figure 4C). Extractable 3‐*O*‐methyltransferase enzymatic activity on caffeoyl‐CoA (for CCoAOMT) and caffeic acid (for COMT) was also induced in ChMYB20‐overexpressing lines (Figure S9a). We then determined lignin content and composition of the hairy roots by thioacidolysis (Figure 4D–I). Lignin content was increased by around 2‐fold in all four ChMYB20‐overexpressing lines (Figure 4F). Expression of ChMYB20 increased the levels of all lignin monomer units, but with a greater effect on S units, thereby resulting in an increase of S/G ratio by 1.2‐ to 2‐fold (Figure 4D, E, G). The percentage of S units was increased in ChMYB20‐overexpressing lines, accompanied by a decrease in G units (Figure 4I). The percentage of H units in lignin from the hairy roots was less than 1% and was only significantly reduced in one of the four independent lines (Figure 4H). The amount of C units was under the limit of quantification. These results confirm ChMYB20 to be a positive regulator of classical G/S lignin biosynthesis, promoting monolignol *O*‐methylation.

C‐lignin biosynthesis correlates with the downregulation of ChMYB20. To determine whether downregulating ChMYB20 could impact lignin composition, we suppressed its expression by RNA interference (RNAi) under the control of the 35S promoter. Sixteen and five independent lines were generated with the ChMYB20‐RNAi or GUS control constructs, respectively (Figure S8b). Four independent lines with 70–95% reduction of ChMYB20 transcript levels were analyzed (Figure 4J). Knockdown of ChMYB20 resulted in a 54–79% reduction in transcript levels of ChCCoAOMTs 1 and 5, respectively, with a log_2_ fold change of −1.11 to −2.25 (Figure 4K). Transcript levels of ChCOMT 1 were also significantly suppressed in ChMYB20 knockdown lines, resulting in a 38–58% reduction with a log_2_ fold change of −0.68 to −1.24, although the transcript levels of ChCOMT2 were unaffected (Figure 4L). We observed an approximately 2‐fold decrease in the amount of G units and a close to 3‐fold reduction in the amount of S units, resulting in a decrease in total lignin thioacidolysis yield of around 2‐fold and a 31–45% reduction of S/G ratio compared with the two GUS control lines (Figure 4N–P). Opposite to the ChMYB20‐overexpressing lines, the percentage of S units decreased in ChMYB20 knockdown lines, accompanied by an increase in G units (Figure 4R). The proportion of H units was again only around 1% and was significantly increased in 3 of the 4 independent lines (Figure 4Q). However, strong downregulation of ChMYB20 was insufficient to cause accumulation of C units, which were again below the level of detection by thioacidolysis. Overall, these results indicate that ChMYB20 regulates total lignin production and S/G ratio in Cleome hairy roots through promotion of monolignol *O*‐methylation.

**Figure 5:**
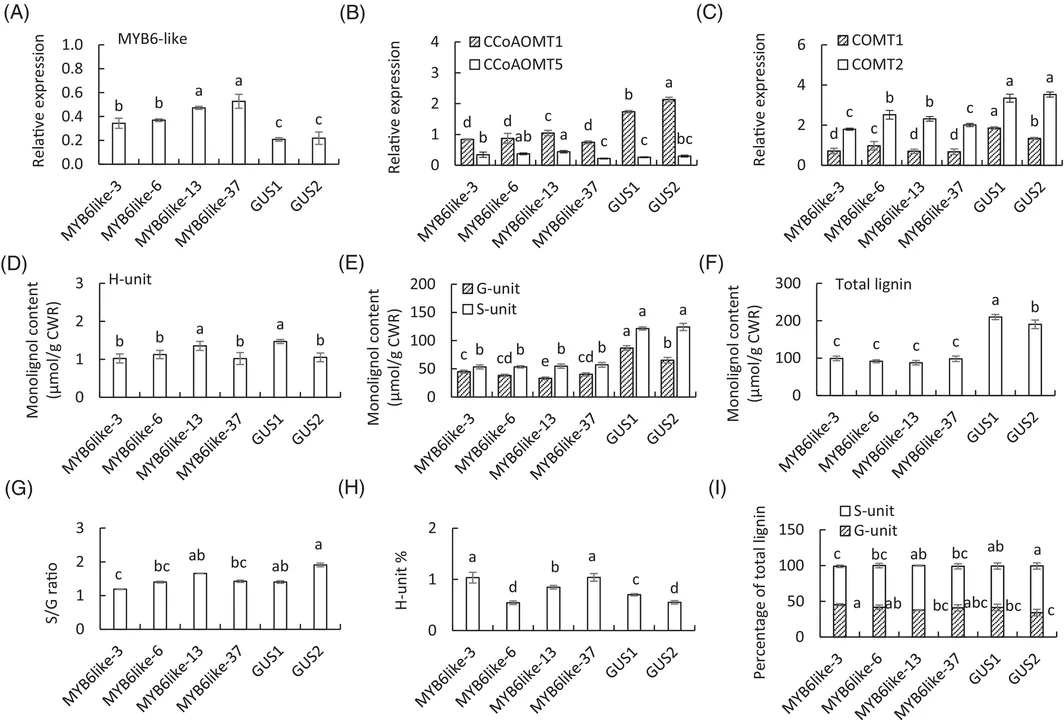
Overexpression of ChMYB6‐like reduces lignin content in *Cleome* hairy roots. (A–C) Transcript levels of ChMYB6‐like (A), ChCCoAOMT1 and ChCCoAOMT5 (b), and ChCOMT1 and ChCOMT2 (C) as determined by qRT‐PCR expressed relative to ubiquitin‐conjugating enzyme E2 11 (*UBC*). (D–F) Lignin monomer thioacidolysis yields for H (d), G and S (E), and total (F) units. CWR, cell wall residue. (G) S/G ratio as determined by thioacidolysis. (H, I) Lignin monomer thioacidolysis yields for H (h) and G and S (i) units as percentages of total lignin units. Four independent lines overexpressing ChMYB6‐like were analyzed and compared with two lines expressing *GUS*. Data are means of three biological replicates. Letters indicate significant differences at the *P* ≤ 0.05 level by one‐way ANOVA (Duncan) with SPSS Statistics.

To test whether ChMYB6‐like can repress ChCOMT and ChCCoAOMT expression *in planta*, we expressed the repressor under the control of the 35S promoter in Cleome hairy roots. Eighteen and five independent lines expressing ChMYB6‐like or GU*S* were generated, respectively (Figure S8c). Four independent lines with 1.6‐ to 2.5‐fold induction of ChMYB6‐like transcript levels were analyzed and compared with two GUS control lines (Figure 5A). Expression of ChMYB6‐like suppressed transcript levels of ChCCoAOMT1 significantly, resulting in a 46–62% reduction with a log_2_ fold change of −0.89 to −1.38, although the transcript levels of ChCCoAOMT5 were unaffected (Figure 5B). However, the transcript levels of ChCCoAOMT1 were as much as ten times higher than those of ChCCoAOMT5 in the GUS control lines (Figure 5B), indicating that *ChCCoAOMT1* is the dominantly expressed *CCoAOMT* gene in Cleome hairy roots. Expression of ChMYB6‐like resulted in a 24–58% reduction of extractable 3‐*O*‐methyltransferase activity with caffeoyl‐CoA compared to GUS control lines (Figure S9b). Expression of ChMYB6‐like suppressed transcript levels of both ChCOMT1 and ChCOMT2 with a log_2_ fold change of −0.45 to −1.24 (Figure 5C), resulting in 16–43% reductions in extractable activity of 3‐*O*‐methyltransferase activity with caffeic acid (Figure S9b). Levels of G and S units and total lignin all decreased following overexpression of ChMYB6‐like (Figure 5E, F), consistent with downregulation of ChMYB20 (Figure 4N, O). However, the effect of ChMYB6‐like overexpression on H units, lignin S/G ratio, and the percentage of each unit was not consistently significant compared with GUS control lines (Figure 5D, G–I).

**Figure 6:**
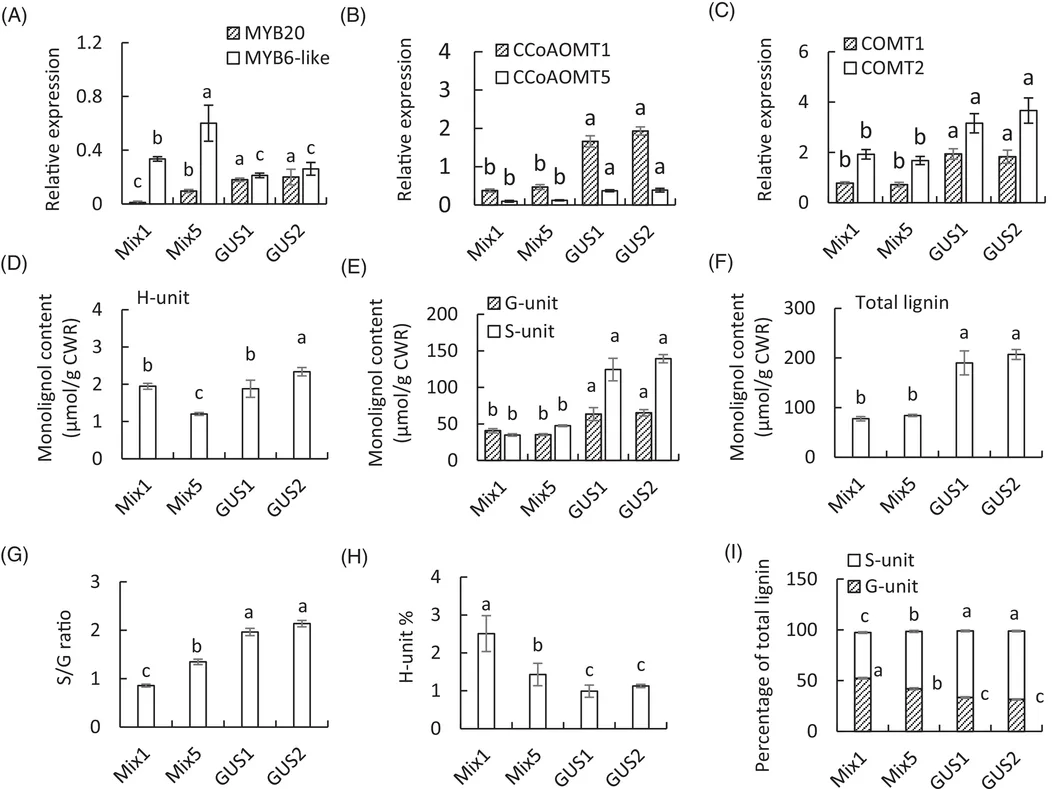
Combination of ChMYB20 knockdown and ChMYB6‐like overexpression alters lignin content and composition in *Cleome* hairy root cultures. (A–C) Transcript levels of ChMYB20 and ChMYB6‐like (A), ChCCoAOMT1 and ChCCoAOMT5 (B), and ChCOMT1 and ChCOMT2 (C) as determined by qRT‐PCR, expressed relative to ubiquitin‐conjugating enzyme E2 11 (UBC). (D–F) Lignin monomer thioacidolysis yields for H (D), G and S (E), and total lignin (F) units. CWR, cell wall residue. (G) S/G ratios as determined by thioacidolysis. (H, I) Lignin monomer thioacidolysis yields for H (H) and G and S (I) units as percentages of total lignin units. Two independent lines with both ChMYB20 knockdown and ChMYB6‐like overexpression constructs were analyzed and compared with two lines expressing GUS. Data are means of three biological replicates. Letters indicate significant differences at the *P* ≤ 0.05 level by one‐way ANOVA (Duncan) with SPSS Statistics.

ChCCoAOMT1 promoter activity was suppressed completely in *in vitro* transactivation assays when the ratio of ChMYB20 and ChMYB6‐like plasmids was the same as the relative transcript ratios of these two genes at the stage when C‐lignin is made in the seed coat (Figure 1C). We therefore hypothesized that similar ratios of the two TFs might likewise suppress OMT expression in hairy roots, potentially resulting in the appearance of C‐lignin. To test this, we co‐transfected the ChMYB20 RNAi construct with the ChMYB6‐like overexpression construct in Cleome hairy roots. From seven independent co‐transformant lines (Figure S8d,e), two lines combined strong reduction of ChMYB20 expression (94% and 50%) with increased ChMYB6‐like expression (1.4‐ and 2.5‐fold; Figure 6A). In these lines, we observed a 68–79% reduction of expression of ChCCoAOMTs 1 and 5 with a log_2_ fold change of −1.64 to −2.25 (Figure 6B) and a 46–62% reduction of expression of ChCOMTs 1 and 2 with a log_2_ fold change of −0.82 to −1.38 (Figure 6C). There was a 59% reduction of total lignin and reduction in the levels of both G and S units, with the greater effect on S units resulting in a 34–58% reduction of S/G ratio compared with GUS control lines (Figure 6E–G). The percentage of S units decreased significantly in these two lines, accompanied by an increase in G units (Figure 6I). The proportion of H units, although still low, was increased in these lines (Figure 6H). The reduction of S/G ratio and the percentage of S units correlated with the reduced transcript levels of ChMYB20. These results indicate that ChMYB6‐like suppression of ChCCoAOMT and ChCOMT expression may operate via competition with ChMYB20. However, the combination of ChMYB20 suppression and ChMYB6‐like expression in these experiments, although indicating control over *O*‐methylation, was still insufficient to support the formation of detectable levels of C units in the lignin.

### Reconstructing gene regulatory networks in cleome hairy roots; the roles of MYB5, MYB42 and NAC92‐like

Loss of lignin *O*‐methylation is accompanied by increased expression of downstream enzymes that may preferentially convert non‐methylated precursors to C‐lignin (Wang et al., 2020; Zhuo et al., 2019). Transient promoter assays above had suggested that ChMYB5 and ChMYB42 were potential regulators of ChCAD5 and ChLAC8 (Figure 2). Based on the same criteria, ChMYB42 was also a potential regulator of ChMYB6‐like, and ChNAC92‐like was a potential regulator of ChMYB42 and ChMYB5 (Figure 3). To test these results *in planta*, we generated sets of transgenic hairy root lines independently overexpressing ChMYB5, ChMYB42 or ChNAC92, with two GUS‐expressing lines included as baseline control. Initially, we tested 20 ChMYB5‐overexpressing lines, 22 ChMYB42‐overexpressing lines, and 24 ChNAC92‐like‐overexpressing lines for determination of transgene transcript levels (Figure S10). In each case, the 8 lines with the highest transgene expression were further selected for determination of lignin content and composition.

**Figure 7:**
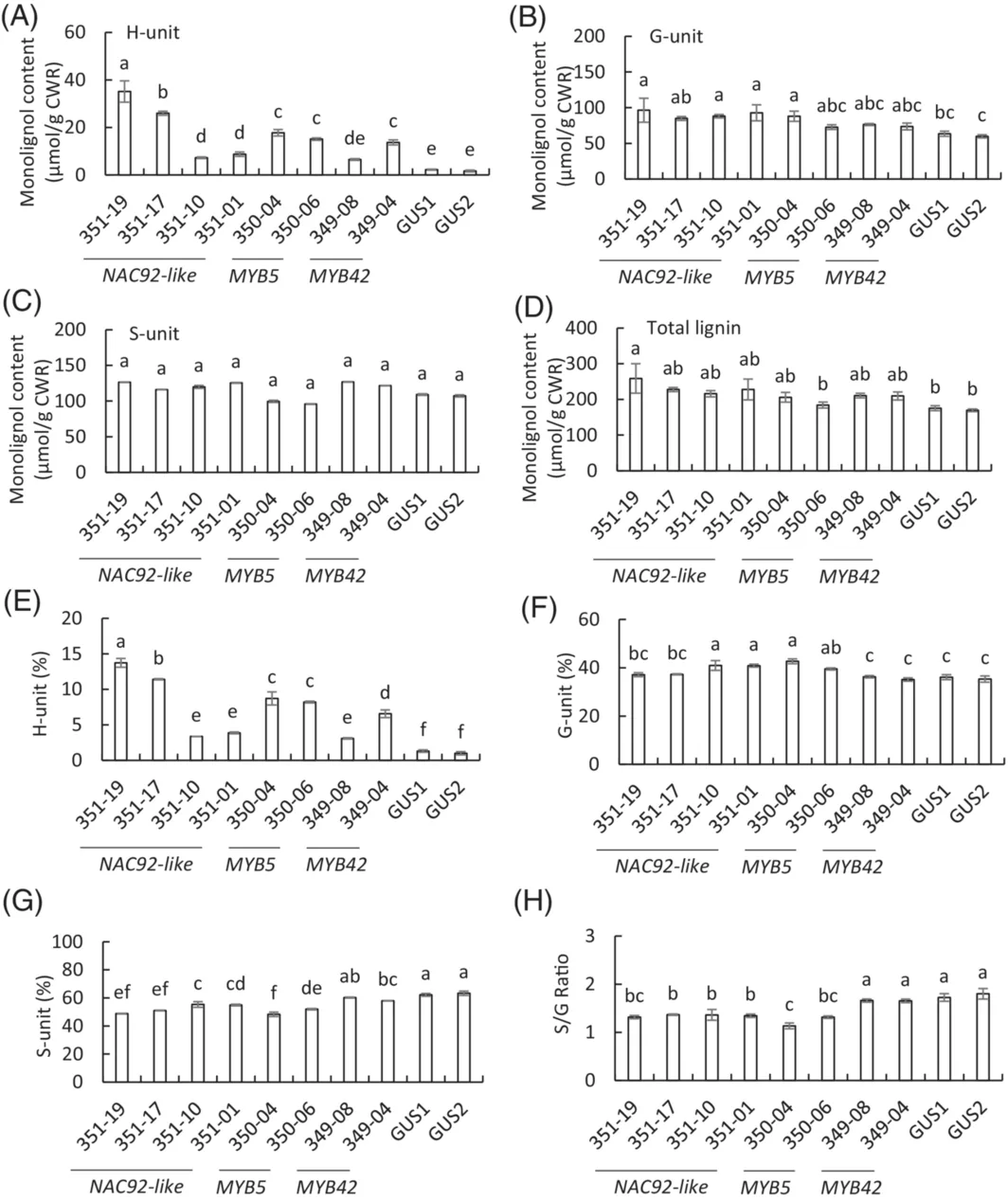
Impacts of overexpression of ChNAC92, ChMYB5 or CMYB42 on lignin content and composition in selected *Cleome* hairy roots. (A–D) Lignin monomer thioacidolysis yields for H (A), G (B), S (C), and total (H + G + S) (D) units. CWR, cell wall residue. (E–G) Lignin monomer thioacidolysis yields for H (E), G (F) and S (G) units as percentages of total lignin units. (H) S/G ratios. Two independent lines with ChMYB5 or ChMYB42 overexpression and four ChNAC92‐like‐overexpressing lines are shown, compared with two lines expressing GUS. Lines 351–19 and 351–17 exhibited 10–15% H units in lignin, whereas lines 351–10 and 351–01 had less than 5% H units. Data are means of three biological replicates. Letters indicate significant differences at the *P* ≤ 0.05 level by one‐way ANOVA (Duncan) with SPSS Statistics.

In the case of ChMYB5 overexpression, all lines except one showed significant increases in the amount and overall % of H units (4.0–7.6‐fold) (Figure S11a,e) but there was no consistent effect on the amounts or percentages of G and S units, although total lignin levels were significantly increased in 4 out of the 8 lines (Figure S11b–d,f–h). A similar increase in H units was seen in lines overexpressing ChMYB42 or ChNAC92‐like, with varying small but inconsistent changes in the percentage of G and S units (Figures S12 and S13). The ChNAC92‐like‐overexpressing lines fell into two groups; one with H units above 10%, the other with H units around 3% (Figure S13e). In none of the experiments reported in Figures S11–S13 were C units in lignin increased above the limit of quantification. The results in Figures S11–S13 are summarized in Figure 7, which highlights ChMYB5‐ and ChMYB42‐overexpressing lines with high H units, and two sets of ChNAC92‐like‐overexpressing lines with different H unit levels.

**Figure 8:**
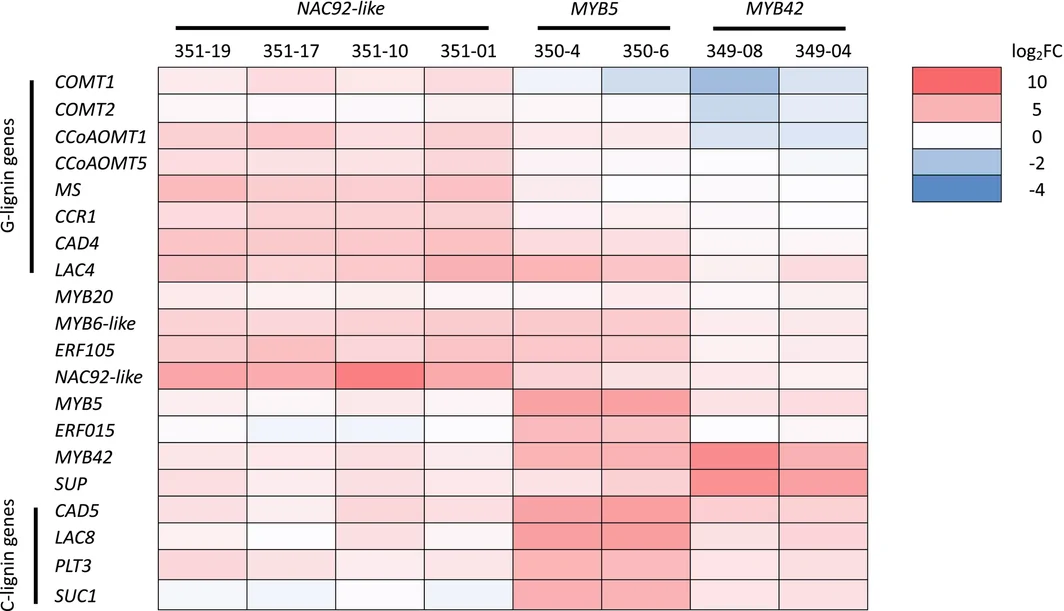
Transcript levels of lignin‐related structural and regulatory genes in *Cleome* hairy roots overexpressing ChNAC92‐like, ChMYB5 or ChMYB42. Two to four TF overexpressing lines were used to check transcript levels of potential target genes. The *Cleome* ubiquitin‐conjugating enzyme E2 11‐like gene was used as an internal standard to normalize the amount of cDNA template. The average transcript levels in the GUS lines were used to calculate fold change. Data are means derived from three biological replicates. Genes with similar expression patterns were grouped together. The table is color‐coded to highlight log_2_ fold change (FC).

To determine whether the changes in lignin content/composition in these lines were related to transcriptional changes of the genes throughout the network, we used qRT‐PCR to interrogate the transcript levels of the structural genes encoding ChCOMTs, ChCCoAOMTs, ChCAD5, and ChLAC8, etc., as well as the identified TFs in the proposed GRN. The results (Figure 8, Table S1) show that overexpression of each TF results in the activation or repression of multiple target genes. In summary, among the structural genes, overexpression of ChMYB42 strongly downregulated the expression of ChCOMT1, ChCOMT2, and ChCCoAOMT1, and induced the expression of ChCAD5, ChLAC8, ChMYB5, ChSUP, ChPLT3 and ChSUC1 (the latter two being transporters implicated in C‐lignin biosynthesis) (Zhuo et al., 2025). These results are consistent with the involvement of ChMYB42 as a positive regulator of the changes that underlie C‐lignin biosynthesis. ChMYB5 overexpression also downregulated ChCOMT1, and very strongly upregulated the ‘late’ C‐lignin pathway structural genes (ChCAD5, ChLAC8, and the two transporters ChPLT3 and ChSUC1) as well as the TFs ChMYB6‐like, ChERF105, ChNAC92‐like, ChMYB42, ChSUP and ChERF015. However, ChCAD4 and ChLAC4, which participate in classical lignin synthesis, were also upregulated in the ChMYB5‐overexpressing lines. Notably, ChNAC92‐like induced genes that are normally downregulated to facilitate C‐lignin synthesis (e.g., C*hCCoAOMT1* and *5*, methionine synthase (*MS*) (Zhuo et al., 2019)), without strongly upregulating genes specific for ‘late stage’ C‐lignin biosynthesis. We did not select the two *ERF* genes, *ChERF015* and *ChERF105*, which activate the ChMYB6‐like and ChMYB42 promoters (Figure 3A, C), for further analysis in this study. However, both were induced by ChMYB5, ChERF015 was also induced by ChNAC92‐like, but neither was induced by ChMYB42 (Figure 8).

## DISCUSSION

### Features of gene regulatory networks for lignin biosynthesis

Several studies have applied GRN analysis to understand the control of lignin biosynthesis under different physiological conditions. For example, the GRN for lignification during poplar wood development was first determined by TF expression in protoplasts coupled with ChIP analysis (Chen et al., 2019). The predicted network consisted of a master regulatory NAC TF (PtrSND1), a middle layer containing MYB TFs, and a 3rd layer of target genes, including several laccases. Further refinement of the model identified a number of additional TFs among the 3rd layer, including MYBs and NACs, with targets including CCoAOMT and CAD. Among these targets, PtrCAD1 was reported to be repressed by PtrMYB174 (Chen et al., 2019). The model developed for transcriptional control of induced lignification in switchgrass cell cultures is similar, with a ‘Layer 1’ NAC transcription factor above two layers of MYB TFs, and one MYB TF (PvMYB4) acting as a repressor (Rao et al., 2019). The model for the regulation of lignin biosynthesis in Arabidopsis vascular tissue also shows multiple layers of transcription factors, where AtMYB20 is regulated by the NAC master switch AtNST1 (Zhong et al., 2007). Knowledge of the transcriptional control of the C‐lignin pathway may provide opportunities to regulate C‐lignin biosynthesis through engineering of one or a few TFs, in a similar way to the engineering of classical lignin (Gui et al., 2019; Zhao et al., 2010), lignans (Kim et al., 2022), alkaloids (Pan et al., 2019) and flavonoids (Dixon et al., 2013; Lu et al., 2017) over recent years.

### ChMYB20 and ChMYB6‐like regulate monolignol *O*‐methylation in cleome

Our studies show that ChMYB20 and ChMYB6‐like act as an inducer and repressor, respectively, of the genes encoding enzymes (COMT and CCoAOMT) catalyzing the two reactions necessary to channel flux in the monolignol pathway away from catechyl (C) units and toward guaiacyl (G) units, with their temporal expression pattern in the Cleome seed coat consistent with this model. This places these two TFs as key components in the regulation of C‐lignin biosynthesis. ChMYB20 has conserved R2R3 domains at its N‐terminal and belongs to the R2R3‐MYB family. R2R3‐MYB TFs have been shown to be involved in secondary metabolism and associated cell development, and those regulating phenylpropanoid synthesis represent one diverse group. They are evolutionarily conserved across different plant species (Chezem & Clay, 2016). The R2R3‐MYB transcription factors have been classified into 28 subgroups according to the conserved motifs at their C‐termini. The lack of such a motif at the C‐terminus of ChMYB20 places it into orphan subgroup 28 (Liu et al., 2015). In Arabidopsis, four members of this subgroup, AtMYB20, AtMYB42, AtMYB43, and AtMYB85, regulate phenylalanine and lignin biosynthesis. The double mutants *myb20/43* and *myb42/85* showed a normal phenotype, whereas the quadruple mutant *myb20/42/43/85* exhibited a semi‐dwarf phenotype with substantial reductions in lignin biosynthesis (Geng et al., 2020). Transcript levels of genes related to lignin biosynthesis were reduced by the simultaneous disruption of MYB20/42/43/85 (Geng et al., 2020). Unlike the genetic redundancy demonstrated among AtMYB20/42/43/85 in Arabidopsis stems, knockdown of ChMYB20 alone significantly altered the lignin composition in Cleome hairy roots. ChMYB20 activated the ChCCoAOMT and ChCOMT promoters in protoplast transactivation assays, and overexpression of ChMYB20 resulted in activation of the *ChCCoAOMT* and *ChCOMT* genes and increased lignin S/G ratio compared with the controls in hairy root lines.

MYB6‐like is classified in the R2R3‐MYB subgroup 4, having R2R3 domains and C1 and C2 motifs. Among the proteins belonging to subgroup 4, some are already known as lignin biosynthesis repressors. Tamagnone et al. (1998) reported the first case, where overexpression of MYB308 and MYB330 from Antirrhinum repressed phenolic acid metabolism and lignin biosynthesis in transgenic tobacco. More recently, the lignin biosynthesis‐associated transcription factor LTF1 knockout mutant of poplar was shown to possess higher lignin content associated with expression of key lignin biosynthesis genes including *COMT2* and *CCoAOMT1* (Gui et al., 2019). In monocots, overexpression of PvMYB4 in transgenic switchgrass resulted in reduced lignin content and ester‐linked p‐coumarate:ferulate ratio and improvement of lignocellulosic feedstocks (Shen et al., 2012).

Our *in vitro* competition data demonstrate the ability of ChMYB6‐like to repress ChMYB20 activity. When ChMYB6‐like was overexpressed along with ChMYB20 knockdown in Cleome hairy roots, the reduction of S/G ratio strongly correlated with the reduced transcript levels of ChMYB20, suggesting that ChMYB6‐like suppression of ChCCoAOMT and ChCOMT expression may operate via competition with ChMYB20. In poplar, the repressor PtrMYB165 can interact with bHLH131, which is part of the MBW complex with the MYB activator, and regulate the flavonoid and phenylpropanoid pathways (Ma et al., 2018). This is not the case for ChMYB20 and ChMYB6‐like, as ChMYB20 lacks the bHLH binding domain. ChMYB6‐like might compete with ChMYB20 by binding to the same regulatory elements; however, such competition could not explain the dramatic suppression of ChMYB20 activation by ChMYB6‐like. It seems more likely that the EAR motif in ChMYB6‐like interacts with the corepressor that recruits the chromatin remodeling complex and represses the target genes. Such recruitments are crucial for regulating genes during development and metabolite synthesis in plants (Baile et al., 2021; Chin et al., 2026; Zheng et al., 2019).

### ChMYB5 and ChMYB42 regulate genes associated with the biosynthesis of C‐lignin

The CAD5 enzyme of Cleome shows a substrate preference for caffealdehyde, and ChLAC8 shows preference for caffeyl alcohol for C‐lignin polymerization (Wang et al., 2020; Zhuo et al., 2019). Both are expressed during the stage of C‐lignin biosynthesis in the seed coat, along with a group of transcription factors, among which ChMYB5 and ChMYB42 were able to transactivate both the ChCAD5 and the ChLAC8 promoters. Overexpression of ChMYB5 and ChMYB42 in Cleome hairy roots strongly upregulated ChCAD5, ChLAC8, ChPLT3, and ChSUC1. The induction of the two latter two proteins, transporters previously implicated in the movement of caffeyl alcohol to the apoplast (Zhuo et al., 2025), provides additional evidence for the function of these transporters. Furthermore, ChMYB5 strongly upregulated ChMYB42, while ChMYB42 also induced ChMYB5 in Cleome hairy roots, suggesting an amplification mechanism.

ChMYB5 is closely related to AtMYB75, which acts as a repressor of the lignin branch of the phenylpropanoid pathway and an inducer of the anthocyanin branch (Borevitz et al., 2000). Similarly, overexpression of grape (*Vitis vinifera*) VvMYB5a and poplar (*Populus tomentosa*) PtMYB6 reduced secondary cell wall deposition and increased anthocyanin and proanthocyanidin accumulation (Deluc et al., 2006; Wang et al., 2019). In our study, the overexpression of ChMYB5 suppressed the transcript level of ChCOMT1, the enzyme which channels flux in the monolignol pathway toward S units, resulting in reduced S unit content and increased H unit content. At the same time, ChMYB5 induced the transcriptional repressor ChMYB6‐like, which inhibited OMT transcription. However, ChMYB5 did not reduce the total lignin content in Cleome hairy roots; it even increased lignin content in several lines, suggesting that ChMYB5 does not act to regulate flux between the lignin and anthocyanin branches as does AtMYB75 in Arabidopsis.

ChMYB42 belongs to the same subgroup of R2R3‐MYB transcription factors as ChMYB20. Overexpression of ChMYB42 also increased the total lignin content in Cleome hairy roots. However, unlike ChMYB20, which induces OMT transcript levels, ChMYB42 suppresses them, resulting in increased H unit content. In Cleome seed coats, ChMYB20 was expressed during the G‐lignin stage, and ChMYB42 was expressed during the C‐lignin stage. ChMYB20 and ChMYB42 therefore play different roles in regulating lignin biosynthesis in Cleome, unlike the genetic redundancy among AtMYB20/42/43/85 in Arabidopsis (Geng et al., 2020).

In Arabidopsis, AtMYB58 and AtMYB63 are well‐known regulators of the lignin pathway (Zhou et al., 2009). Phylogenetic analysis showed that Arabidopsis MYB58/MYB63 and ChMYB20/ChMYB42 identified in this study belong to the activator clade. However, they fall into different subgroups, suggesting that distinct MYBs regulate lignin biosynthetic genes in different tissues and at different developmental stages. Although ChMYB42 expression represses lignin *O*‐methylation, it does so through activation of the ChMYB6‐like repressor. Thus, the phylogenetically related ChMYB20 and ChMYB42 can act in opposite directions while both being transcriptional activators. ChMYB42 acts as a dual function switch by directly activating C‐lignin‐specific processes and indirectly repressing G‐lignin‐specific processes in the Cleome seedcoat.

### The role of ChNAC92‐like

NAC transcription factors act as the master regulators in lignin biosynthesis (Chen et al., 2019; Rao et al., 2019; Zhong et al., 2007). Our studies showed that ChNAC92‐like upregulated the ChMYB5 and ChMYB42 promoters *in vitro*, suggesting that it might function one tier above these transcription factors in the C‐lignin regulatory circuit. However, overexpression of ChNAC92‐like in Cleome hairy roots did not consistently activate the module of genes associated with C‐lignin biosynthesis; rather, it activated genes encoding enzymes that are necessary for regular G/S lignin formation such as OMTs, methionine synthase (MS), CCR1, CAD4 and LAC4 (Zhuo et al., 2019), irrespective of whether the hairy root lines had high or intermediate levels of H units. ChNAC92‐like is closely related to Arabidopsis ANAC087 (At5G18270). Overexpression of ANAC087 induced leaf senescence and anthocyanin accumulation (Vargas‐Hernández et al., 2022). The effects of ANAC087 on lignin content are unknown, although it is expressed in the vasculature and stem (Vargas‐Hernández et al., 2022).

In Arabidopsis, AtNST1 and AtNST2 function redundantly in activating secondary cell wall biosynthesis in anther endothecium, and their simultaneous mutations cause a defect in anther dehiscence (Mitsuda et al., 2005). In fibers, AtNST1 functions redundantly with AtSND1, secondary wall‐associated NAC domain protein1, and their double mutation results in a loss of secondary cell wall thickening (Zhong et al., 2007). Mutation of *NST1* results in a reduction of expression of AtMYB20 (Zhong et al., 2008), indicating that AtMYB20 is a downstream transcription factor of AtNST1. In our transactivation assays using Arabidopsis protoplasts, we observed that the homolog of AtNST1, ChNAC43, could weakly activate all four ChCCoAOMT and ChCOMT promoters. Similarly, ChNAC43 may regulate the ChCCoAOMT and ChCOMT promoters via ChMYB20.

Future studies will need to target ChNAC92‐like and ChNAC43 and other TF genes directly in the Cleome seed coat by gene editing; useful as *in vitro* transactivation and hairy root transformation studies are, they lack the cellular context of the developing seed coat, which may be the reason for the different network positioning of ChNAC92‐like based on *in vitro* transactivation and hairy root assays.

### Why was no C‐lignin detected in cleome hairy roots?

No C‐lignin was observed in any of the above experiments in which TF levels were modulated in Cleome hairy roots. Previous studies have indicated that C units can be detected, up to 20% of total lignin units, in *Medicago truncatula* hairy roots only if CCoAOMT is very strongly downregulated in a *comt* mutant genetic background (Ha et al., 2023). We assume that, although both ChCOMT and ChCCoAOMT downregulation occurs on overexpression of the upstream TFs ChMYB5 and particularly ChMYB42, residual OMT activity precludes C unit formation in the Cleome hairy roots. An alternative explanation is that additional factors may limit C‐lignin accumulation in Cleome hairy roots as compared to the developing seed coat. For example, although overexpression of ChMYB5 and ChMYB42 upregulated the transporters ChPLT3 and ChSUC1, previously implicated in C‐lignin biosynthesis (Zhuo et al., 2025), the exact expression pattern of these transporters and their activity levels is not known. Furthermore, competing pathways for utilization of caffeic acid may be present in hairy roots.

Total lignin was strongly reduced, via reductions in both G and S units, when ChMYB20 and ChMYB6‐like levels were engineered in an attempt to mimic the situation in the Cleome seed coat at the onset of C‐lignin biosynthesis, but, in addition to the lack of C units, there was no significant change in levels of H units. In contrast, overexpression of the ‘upper layer’ TFs ChNAC92‐like, ChMYB5 or ChMYB42 resulted in little effect on total lignin level, but a significant increase in H units. It is possible that different fates of coumaroyl CoA determine the extent of accumulation of H units in these experiments.

### Models for the control of C‐lignin biosynthesis in the cleome seed coat

**Figure 9:**
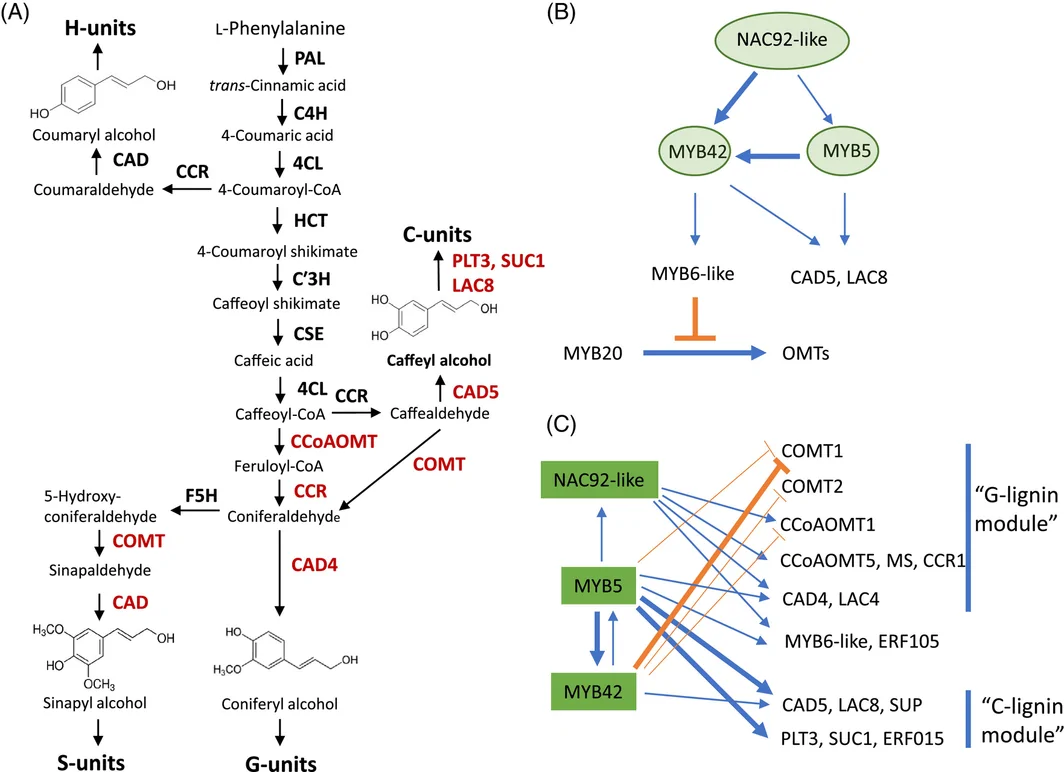
Models for regulatory networks underlying switches in lignin composition in *Cleome*. (A) Diagram of the pathways to H, C, G and S lignins. Enzymes in red are targets of the TFs described in this paper and are defined in the text. (B) Network based on promoter assays in Arabidopsis protoplasts. Thin arrows indicate regulation with relative fold change >5 for TFs or >10 for lignin pathway genes. Thicker arrows indicate regulation with relative fold change >10 for TFs or >20 for lignin genes. Bar indicates repression. (C) Network based on qRT‐PCR analysis of transgenic *Cleome* hairy roots independently overexpressing the transcription factors in green boxes. Thin arrows indicate regulation with relative log_2_ fold change >2 or <−0.5. Thicker arrows/barred lines indicate regulation with relative log_2_ fold change >4 or <−1. In panels (B) and (C), blue arrows represent activation, orange arrows repression.

Figure 9 presents a schematic for the biosynthetic pathways to all the four lignin building blocks (a), and two versions of models for the network regulating the lignin biosynthesis pathway based on Arabidopsis protoplast transactivation assays (b) and transgenic Cleome hairy root verification (c), respectively. Note that the seed coat only naturally accumulates G‐ and C‐lignins (temporally separated), whereas the hairy roots can make lignins with H, G, and S units. The models share many of the features determined for transcriptional control of lignin biosynthesis in other species; in panel b, based only on the transactivation assays, Layer 1 is represented by NAC92‐like, Layer 2 by MYB42 and MYB5, and Layer 3 by MYB6‐like. However, because C‐lignin biosynthesis requires the cessation of part of the monolignol pathway (the OMT reactions) while at the same time needing continued flux through the later stages, the network possesses an additional layer, represented by MYB20, the activity of which is suppressed by MYB6‐like to turn off COMT and CCoAOMT expression. The model in Figure 9B explains how NAC92‐like could set into play a cascade resulting in the activation of C‐lignin synthesis at the expense of G‐lignin biosynthesis in the seed coat. During development of the Cleome seed coat, ChMYB20 is expressed early and downregulated around 12 DAP, whereas expression of ChMYB6‐like is elevated from around 12 DAP. The function of ChMYB6‐like at the latter stage may be to ensure full repression of OMT expression via the MYB20 pathway, and possibly to compete with other MYB activators to maintain the repression of the *ChCCoAOMT* and *ChCOMT* genes. Notably, its expression is regulated by the same TF, MYB42, that activates expression of downstream genes for C‐lignin synthesis.

An alternative approach to understanding GRNs is to follow the consequences of up‐ or downregulation of TFs in transgenic plant tissues (e.g., Rao et al., 2019). The model in Figure 9C is based upon the determination of transcript levels of potential TF targets in Cleome hairy roots following upregulation of ChNAC92‐like, ChMYB5 or ChMYB42 (Figure 8), and the data provide confirmation for several features of the scheme in Figure 9B. The broad features of the model are that overexpression of MYB42 leads to downregulation of COMT1, COMT2 and CCoAOMT1 (the ‘G‐lignin module’), along with upregulation of LAC8, CAD5, PLT3 and SUC1 (the ‘C‐lignin module’). Induction of C‐lignin genes by MYB42 may operate indirectly through activation of SUP and ERF015. Similarly, overexpression of MYB5 leads to downregulation of COMT1 and upregulation of C‐lignin genes. This dual activity and the amplification mechanism between ChMYB42 and ChMYB5 likely make them critical players in the switch from G‐ to C‐lignin biosynthesis. Further experimentation will be necessary to define the roles of NAC92‐like, ERF015, and SUP (SUPERMAN), which is a floral homeotic gene (Yun et al., 2002).

In conclusion, transactivation and TF overexpression analyses support a model for monolignol *O*‐methylation being controlled by the relative levels of MYB20 and MYB6‐like in Cleome, with additional MYB and NAC TFs comprising a second layer of regulation. Further testing of this model will require analysis of seed coat composition in transgenic Cleome with gain or loss of function of the TFs identified in this work. This may possibly reveal additional transcriptional control of C‐lignin that was missed in the present study. The ultimate goal is to enable the engineering of this novel polymer in quantities that will allow its full exploitation for the production of valuable chemicals and biomaterials.

## MATERIALS AND METHODS

### Chemicals

Caffeic acid was purchased from Sigma‐Aldrich, USA. Caffeoyl‐CoA was obtained from MicroCombichem, Germany.

### Plant materials

*Cleome hassleriana* plants were grown in a greenhouse set at a 16‐h/8‐h day/night cycle at 24–28°C. The flowers were hand pollinated. Mature seeds were harvested, dried at 35°C for 1 week, and kept at 4°C in a cold room until needed.

### WGCNA network construction

RNA‐Seq datasets sourced from the seed coats of *C. hassleriana* (Cleome) were downloaded from the NCBI Sequence Read Archive and aligned to the ASM46358v1 transcriptome (Cheng et al., 2013) via salmon v1.1.0. Log_2_ normalized expression values (log_2_ TPM) for each transcript across all datasets were used to generate a coexpression network using the WGCNA package (Langfelder & Horvath, 2008). Transcripts with zero expression in more than half of the datasets were removed from downstream network construction to minimize their impact on the correlations upon which each network is founded. A soft‐thresholding power of 8 was used to construct unsigned networks with a specified minimum module size of 25. Network generation was primarily carried out by WGCNA's Block Wise Modules function with Deep Split and Merge Cut Height parameters set to 4 and 0.25, respectively. Hierarchical cluster maps and scatterplots were built using the Seaborn 0.10.1 cluster map and scatter plot functions. Sample‐wise hierarchical clustering dendrograms were built using R v3.5.3. Additional modifications, such as the cluster map sample legend, were manually constructed using Inkscape v0.92.

### Identification of modules associated with lignin biosynthesis

The lignin genes in Cleome (Zhuo et al., 2019) were collated, and the modules associated with them were identified and listed in Table 1. Module transcript memberships were extracted for each network constructed using WGCNA for further sub‐clustering using a Markov clustering strategy (MCL). First, the log_2_ normalized expression (log_2_ TPM) of all transcripts in a particular module was used to calculate the Pearson correlation coefficient for each of the transcript pairs. An initial hard‐threshold of 0.75 and a stricter secondary hard‐threshold of 0.90 were used to isolate the strongest coexpression relationships within the module, followed by clustering using the MCL algorithm at increasing inflation parameters ranging from 1.4 to 8. To obtain an appropriate size of the TF candidate pool, an inflation factor of 1.8 was used to subcluster Module 1. For Modules 2 and 4, there were only a few TFs, so no sub‐clustering was needed. To identify additional TF candidates associated with CAD5 and LAC8, TFs with a correlation coefficient greater than 0.80 were included.

### Genomic DNA and RNA isolation

Genomic DNA was extracted from young leaves using a hexadecyltrimethylammonium bromide (CTAB) method (Murray & Thompson, 1980). Total RNA was isolated using TRI Reagent (Invitrogen, Waltham, MA, USA) according to the manufacturer's protocol.

### Quantitative reverse transcription polymerase chain reaction (qRT‐PCR)

First‐strand cDNA was synthesized from total RNA using the SuperScript III First‐Strand Synthesis System (Invitrogen) after treatment with DNase I (Invitrogen). qRT‐PCR was conducted with a QuantStudio 6 Flex Real‐Time PCR System (Applied Biosystems, Waltham, MA, USA). Primers are listed in Table S2. The primer specificity was validated by melting profiles showing a single product at a specific melting temperature. Parallel reactions to amplify the ubiquitin‐conjugating enzyme E2 11‐like gene (*UBC*) were used to normalize the amount of template. Relative transcript levels were calculated using the formula for comparative Ct value (Ranasinghe et al., 2008) with three biological replicates.

### Promoter transactivation assays

According to the genome sequences at NCBI, 1–1.5 kb promoter regions of target genes were PCR amplified from Cleome genomic DNA using the primer pairs listed in Table S2. Construction of promoter‐reporter constructs in the modified p2GW7 vector (Karimi et al., 2002) was performed as described (Zhao et al., 2010). The coding regions of the test transcription factors were amplified from pooled Cleome seed coat cDNA (Zhuo et al., 2019) using the primers shown in Table S2 and cloned into pENTR/D‐TOPO vector (Invitrogen) and then introduced into p2GW7 by LR reaction (Invitrogen) to obtain the effector constructs. Arabidopsis protoplasts were isolated and transformed as described by Ferreira and Antunes (2024). A Renilla luciferase gene was also cloned into p2GW7 to form the reference construct to determine the transfection efficiency. The luciferase activities were determined using the dual‐luciferase reporter assay system (Promega, Madison, WI, USA) according to the manufacturer's instructions and read by a GloMax 96 Microplate Luminometer (Promega). The firefly luciferase activity was calculated by normalizing against the Renilla luciferase activity in each transfection event. Data are presented as averages ± SD over the three biological replicates.

### Phylogenetic analysis

MYBs that regulate secondary cell wall biosynthesis or phenylpropanoid metabolism were identified from the literature (Liu et al., 2015; Ma & Constabel, 2019; Zhao & Dixon, 2011; Zhong et al., 2010). Protein sequences were obtained from the NCBI protein database. Using the MEGA (Molecular Evolutionary Genetics Analysis version 11, Tamura et al., 2021) software, first the multiple protein sequence alignment was performed by the ClustalW program under the default setting, and then the phylogenetic tree was constructed using the Neighbor‐Joining method (Saitou & Nei, 1987) with 1000 bootstrap replicates.

### Expression of targeted transcription factors in cleome hairy roots

To overexpress TFs, the ORFs of *ChMYB20*, *ChMYB6‐like, ChMYB42, ChMYB5*, and *ChNAC92‐like* within the pENTR/D‐TOPO vector (Invitrogen) were subcloned into the pB7WG2D binary vector driven by the CaMV 35S promoter via LR recombination reaction (Invitrogen). To knock down *ChMYB20*, a fragment of it was amplified using the primers shown in Table S2, cloned into pENTR/D‐TOPO vector (Invitrogen), then subcloned into p7GWIWG2(I) binary vector driven by the CaMV 35S promoter via LR recombination reaction (Invitrogen). The resulting plasmids were transformed into *Agrobacterium rhizogenes* ARqual. *Agrobacterium*‐mediated Cleome hairy root transformation was performed as described previously (Liu et al., 2014). To harvest hairy root samples, sections 3 cm from the root tip were collected at 2 weeks after subculture. Samples were collected from three individual plates for each individual line and immediately frozen in liquid nitrogen, then stored at −80°C prior to analysis.

### Assay of enzyme activities in protein extracts from hairy roots

Hairy root samples were collected and ground to a fine powder. About 0.3 g of tissues was suspended in 1 mL of extraction buffer, and protein extracts were obtained and quantified as described previously (Zhuo et al., 2019). Twenty μL of protein extracts (6–11 μg) were incubated in a 200 μL mixture with 50 μM substrate, and assay of COMT and CCoAOMT activities by HPLC separation and quantification of reaction products was performed as described previously (Zhuo et al., 2019) with three biological replicates.

### Determination of lignin composition

Hairy root samples were extracted sequentially with methanol, chloroform/methanol (2:1, v/v), methanol and water, and then lyophilized. Lignin composition was determined by thioacidolysis (Chen et al., 2021). Lignin monomers were quantified using 4,4′‐ethylidenebisphenol as an internal standard. Three biological replicates were measured.

### Statistical analysis

Comparisons were made using Duncan grouping at the 0.05 probability level or an unpaired two‐tailed *t*‐test with SPSS statistics (version 29 by IBM).

## Author Contributions

CZ, DB, XX, XR, FC performed experiments; CZ, RKA, FC, XR and RAD analyzed data, CZ and RAD wrote the manuscript.

## Conflict of Interest

None declared.

## Supporting information


**Figure S1.** Sample‐to‐sample Pearson correlation map used in the construction of the WGCNA‐derived network. Dendrograms represent the hierarchical clustering of samples based on average euclidean distance. Perimeter boxes indicate the dataset of each individual sample.
**Figure S2**. Scatterplot of alignment statistics based on the Salmon pseudoalignment of all 18 datasets used in the construction of the WGCNA‐derived network to the *C. hassleriana* transcriptome (ASM46358v1, Cheng et al., 2013). Vertical position reflects the percentage of reads successfully aligned per each dataset, and horizontal position reflects the percentage of ASM46358v1 transcripts represented per each dataset. Node size is proportional to the size of each dataset. Because the available *C. hassleriana* transcriptome was generated from leaf tissue, whereas our RNA‐seq data were generated from seed coats, some seed‐specific transcripts were not represented in the reference transcriptome and thus became unmapped reads.
**Figure S3**. Hierarchical clustering of datasets used in the construction of the WGCNA‐derived network after filtering. Distance is based on the Euclidean distance across sample‐to‐sample Pearson correlations. Label colors represent the dataset of each individual sample. The height axis displays the distance between clusters. The bars indicate the point at which two clusters are merged.
**Figure S4**. ChMYB5 does not synergize with ChNAC92‐like or AtTTG1/TT8 to activate the promoter of ChMYB42. The promoter of ChMYB42 was cloned and linked to the reporter firefly luciferase. The test transcription factors were cloned and constructed under the control of the 35S promoter in the effector vector. Together with the internal control plasmids, they were transfected to the Arabidopsis protoplasts. Dual‐luciferase reporter assay was performed after 20 h of incubation. Data are means of duplicate measurements of three biological replicates. The asterisks indicate significant differences at the *P* ≤ 0.05 level by unpaired two‐tailed *t*‐test.
**Figure S5**. Phylogenetic analysis of ChMYB20, ChMYB6‐like, ChMYB5 and ChMYB42 together with other MYBs known to regulate secondary wall biosynthesis or phenylpropanoid metabolism. Protein sequences were obtained from GenBank. The phylogenetic tree was constructed using the Neighbor‐joining method (Saitou & Nei, 1987) with 1000 bootstrap replicates by the software MEGA 11 (Molecular Evolutionary Genetics Analysis version 11, Tamura et al., 2021). Numbers indicate confidence percentages. Blue indicates the activator clade, orange indicates the repressor clade, purple indicates the third clade.
**Figure S6**. Multiple sequence alignments of ChMYB20 and ChMYB42 (a), ChMYB6‐like (b) and ChMYB5 (c) with closely related MYBs.
**Figure S7**. cis‐Element analysis of ChCCoAOMTs, ChCOMTs, ChCAD5, and ChLAC8 promoters. A square represents AC elements with the consensus sequences ACCTNNC (Mellway et al., 2009) or secondary wall MYB‐responsive elements (SMRE) with the consensus sequences ACC(A/T)A(A/C)(T/C) (Zhong & Ye, 2012) in promoter regions, and numbers indicate the position of these motifs relative to the translational start (marked by an arrow).
**Figure S8**. Transgene transcript levels in *Cleome* hairy roots overexpressing ChMYB20, ChMYB6‐like and knockdown of ChMYB20. Transcript levels of ChMYB20 (a, b, d) or ChMYB‐like (c, e) in transgenic *Cleome* hairy roots overexpressing ChMYB20 (a), knockdown of ChMYB20 (b), overexpressing ChMYB6‐like (c), and with the combination of knockdown of ChMYB20 and overexpressing ChMYB6‐like (d, e). Transcript levels were determined by qRT‐PCR and expressed relative to ubiquitin‐conjugating enzyme E2 11 (*UBC*). Data are means of three biological replicates.
**Figure S9**. Effect of expression of ChMYB20 or ChMYB6*‐like* on 3‐*O*‐methyltransferase enzymatic activity in *Cleome* hairy roots. Extractable 3‐*O*‐methyltransferase enzymatic activity on caffeoyl‐CoA (for CCoAOMT) and caffeic acid (for COMT) in *Cleome* hairy root cultures overexpressing ChMYB20 (a) or ChMYB6‐like (b). Four independent lines overexpressing ChMYB20 or ChMYB6‐like were analyzed and compared with two lines expressing GUS. Data are means of three biological replicates. Letters indicate significant differences at the *P* ≤ 0.05 level by one‐way ANOVA (Duncan) with SPSS Statistics.
**Figure S10**. Transcript levels of transcription factors in *Cleome* hairy roots overexpressing ChMYB5 (a), ChMYB42 (b) or ChNAC92‐like (c), as determined by qRT‐PCR. The *Cleome* ubiquitin‐conjugating enzyme E2 11‐like gene was used as an internal standard to normalize the amount of cDNA template. The average transcript levels in the GUS lines was set as 1. The top eight lines with the highest transcript levels were selected for lignin assays by thioacidolysis.
**Figure S11**. Lignin content and composition in *Cleome* hairy roots overexpressing ChMYB5. (a–d) Total monomer units and lignin amount as determined by thioacidolysis. CWR, cell wall residue. (e–g) Percentage of H, G and S units in the above lignin samples. (h) S/G ratio.
**Figure S12**. Lignin content and composition in *Cleome* hairy roots overexpressing ChMYB42. (a–d) Total monomer units and lignin amount as determined by thioacidolysis. CWR, cell wall residue. (e–g) Percentage of H, G and S units in the above lignin samples. (h) S/G ratio.
**Figure S13**. Lignin content and composition in *Cleome* hairy roots overexpressing ChNAC92‐like. (a–d) Total monomer units and lignin amount as determined by thioacidolysis. CWR, cell wall residue. (e–g) Percentage of H, G and S units in the above lignin samples. (h) S/G ratio.
**Table S1**. Fold change of lignin‐related structural and regulatory gene transcript levels in *Cleome* hairy roots overexpressing ChNAC92‐like, ChMYB5 or ChMYB42 Two to four TF overexpressing lines were used to check transcript levels of potential target genes. The *Cleome* ubiquitin‐conjugating enzyme E2 11‐like gene was used as an internal standard to normalize the amount of cDNA template. The average transcript levels in the GUS lines were used to calculate fold change. Data are means derived from three biological replicates. Genes with similar expression pattern were grouped together. The log_2_ fold change is visualized as a Heatmap in Figure 8.
**Table S2**. Primers used in the present work.

## Data Availability

The data sets supporting the results of this article are available in the NCBI Sequence Read Archive (SRA) with accessions SRX4923580‐SRX4923609.
