# Supplementary material for: Transcriptional regulation of monolignol O‐methylation in Cleome hassleriana
Source: Plant J. 2026 Sep 21;127(6):e71123. doi: 10.1111/tpj.71123 (PMC13592876; doi:10.1111/tpj.71123)
Supplement: Supplementary file 1 [file TPJ-127-0-s001.pdf]

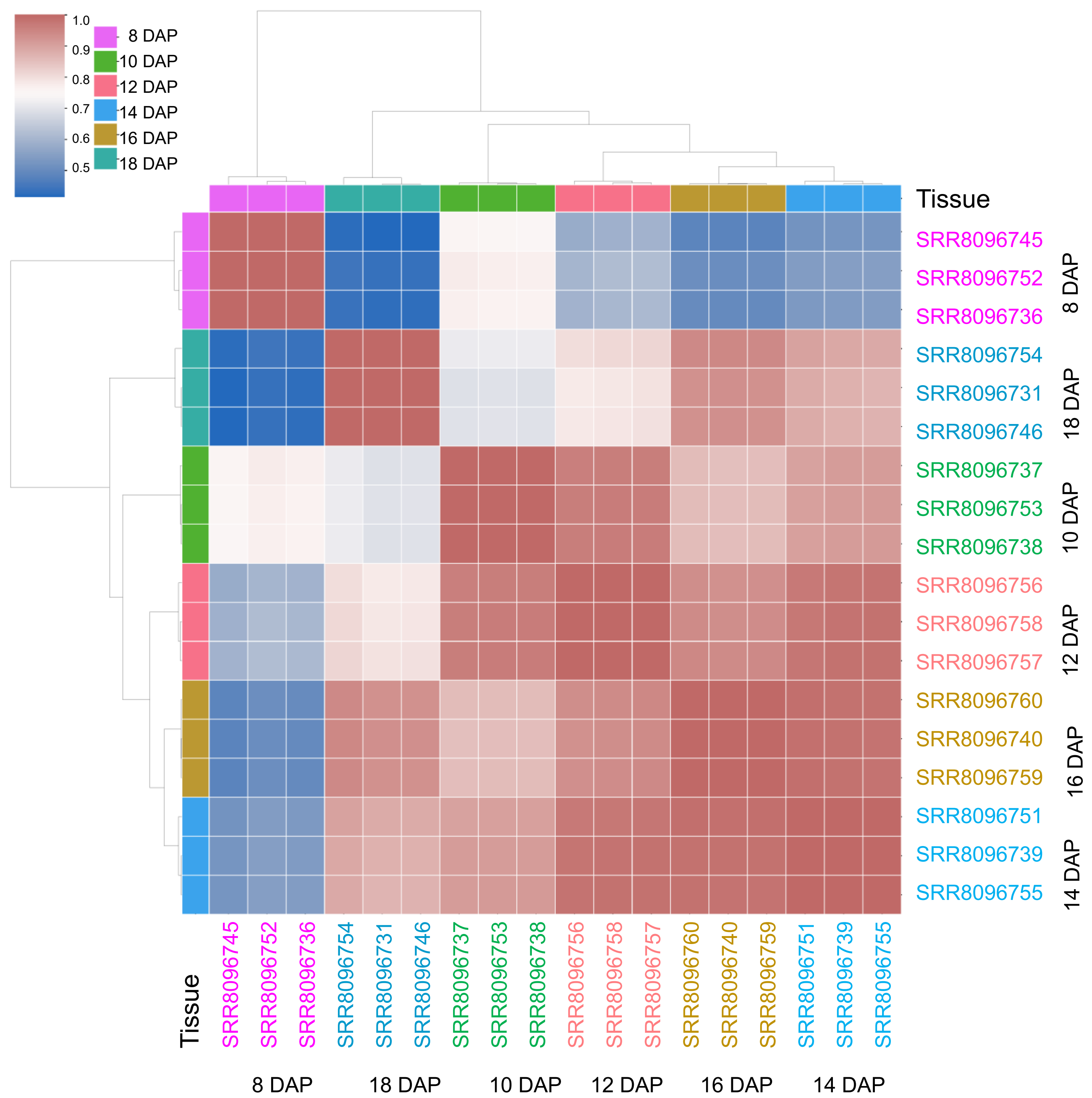

Figure S1. Sample to sample Pearson correlation map used in the construction of the WGCNA-derived network. Dendrograms represent the hierarchical clustering of samples based on average euclidean distance. Perimeter boxes indicate the dataset of each individual sample.

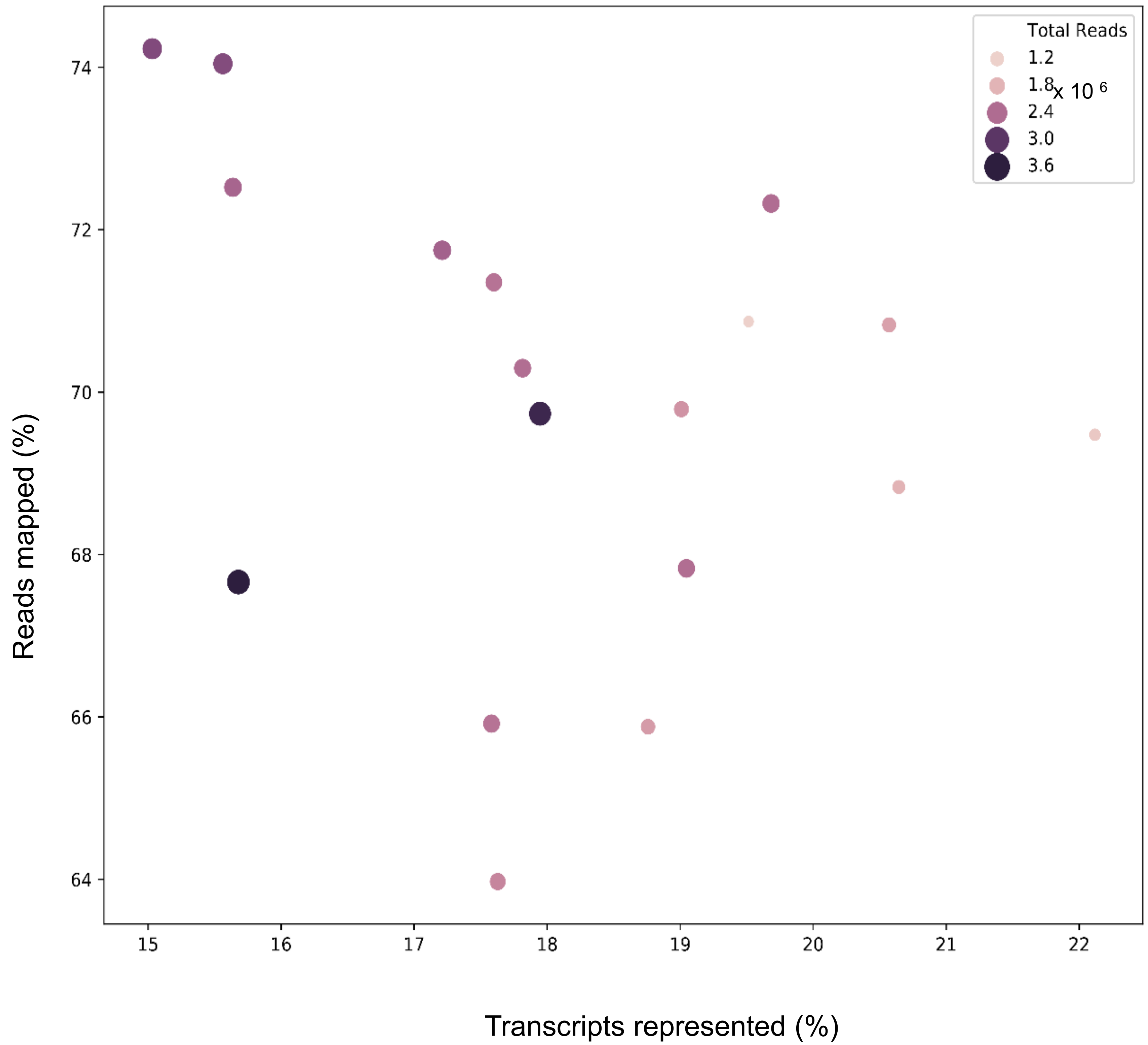

Figure S2. Scatterplot of alignment statistics based on the Salmon pseudoalignment of all 18 datasets used in the construction of the WGCNA-derived network to the *C. hassleriana* transcriptome (ASM46358v1, Cheng et al., 2013). Vertical position reflects the percentage of reads successfully aligned per each dataset, and horizontal position reflects the percentage of ASM46358v1 transcripts represented per each dataset. Node size is proportional to the size of each dataset. Because the available *C. hassleriana* transcriptome was generated from leaf tissue, whereas our RNA-seq data were generated from seed coats, some seed-specific transcripts were not represented in the reference transcriptome and thus became unmapped reads.

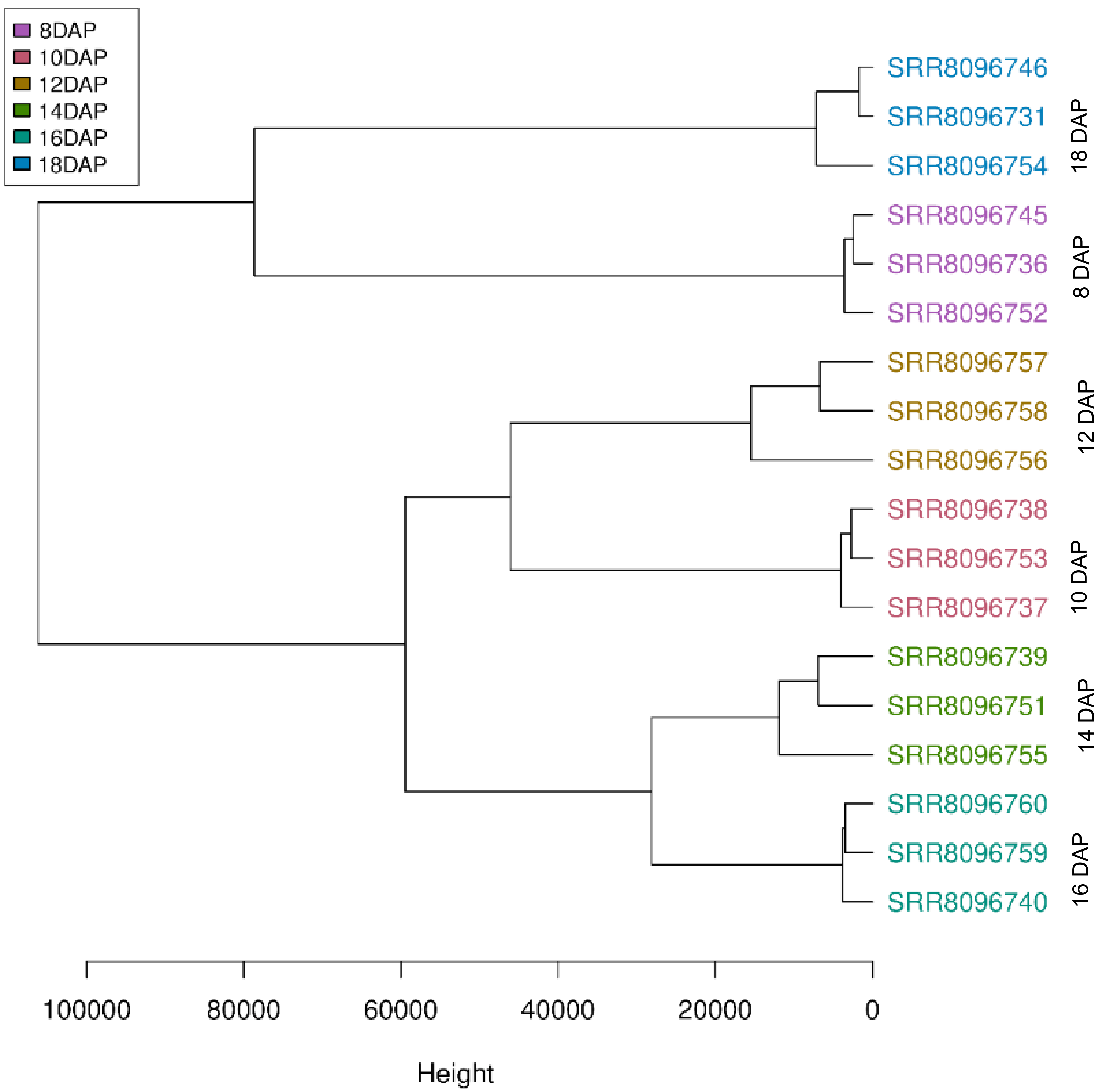

Figure S3. Hierarchical clustering of datasets used in the construction of the WGCNA-derived network after filtering. Distance is based on the Euclidean distance across sample-to -sample Pearson correlations. Label colors represent the dataset of each individual sample. The height axis displays the distance between clusters. The bars indicate the point at which two clusters are merged.

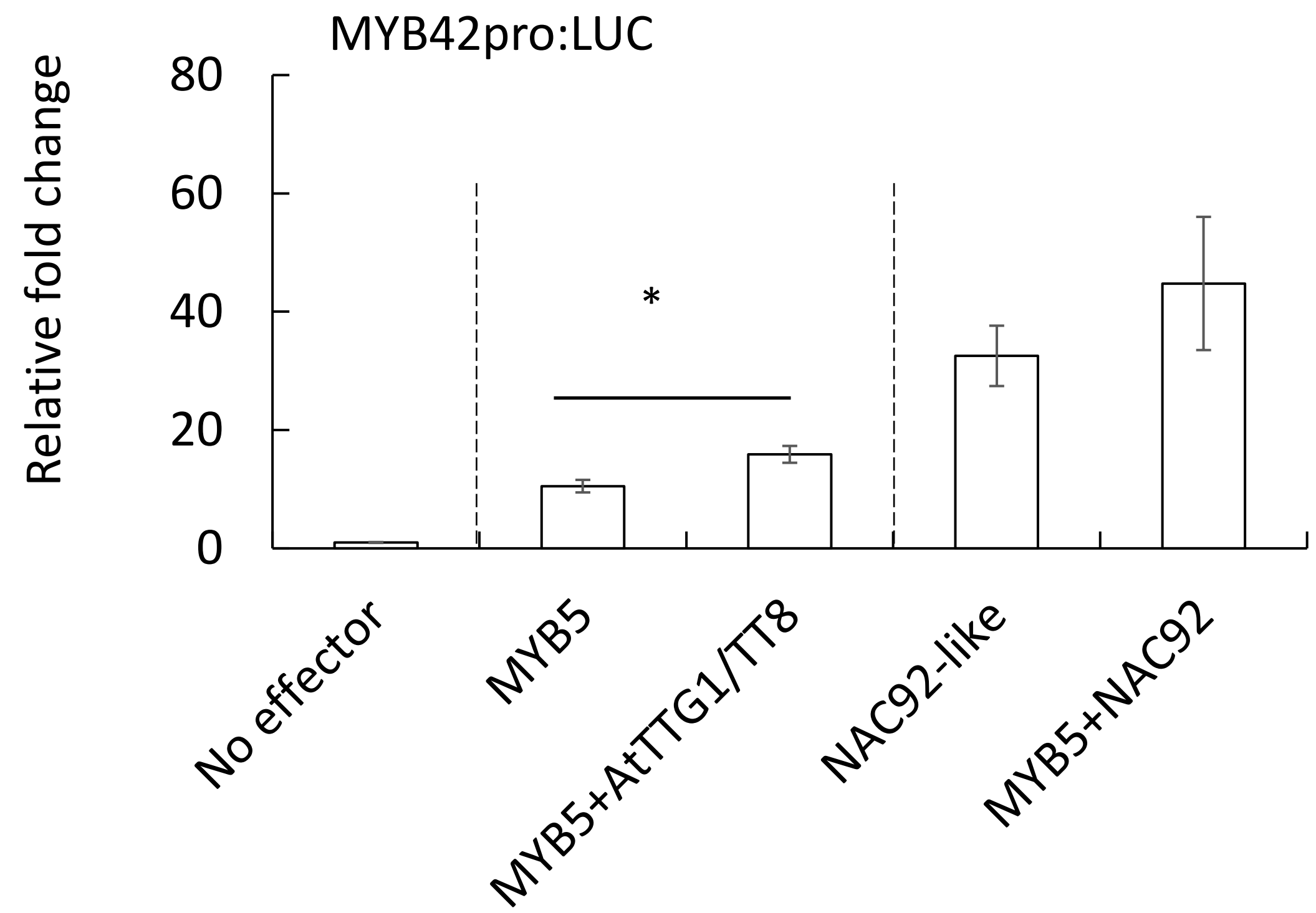

Figure S4. ChMYB5 does not synergize with ChNAC92 or AtTTG1/TT8 to activate the promoter of ChMYB42.
The promoter of ChMYB42 was cloned and linked to the reporter firefly luciferase. The test transcription factors were cloned and constructed under the control of the 35S promoter in the effector vector. Together with the internal control plasmids, they were transfected to the *Arabidopsis* protoplasts. Dual-luciferase reporter assay was performed after 20 h of incubation. Data are means of duplicate measurements of three biological replicates. The asterisks indicate significant differences at the $P \leq 0.05$ level by unpaired two-tailed t-test.

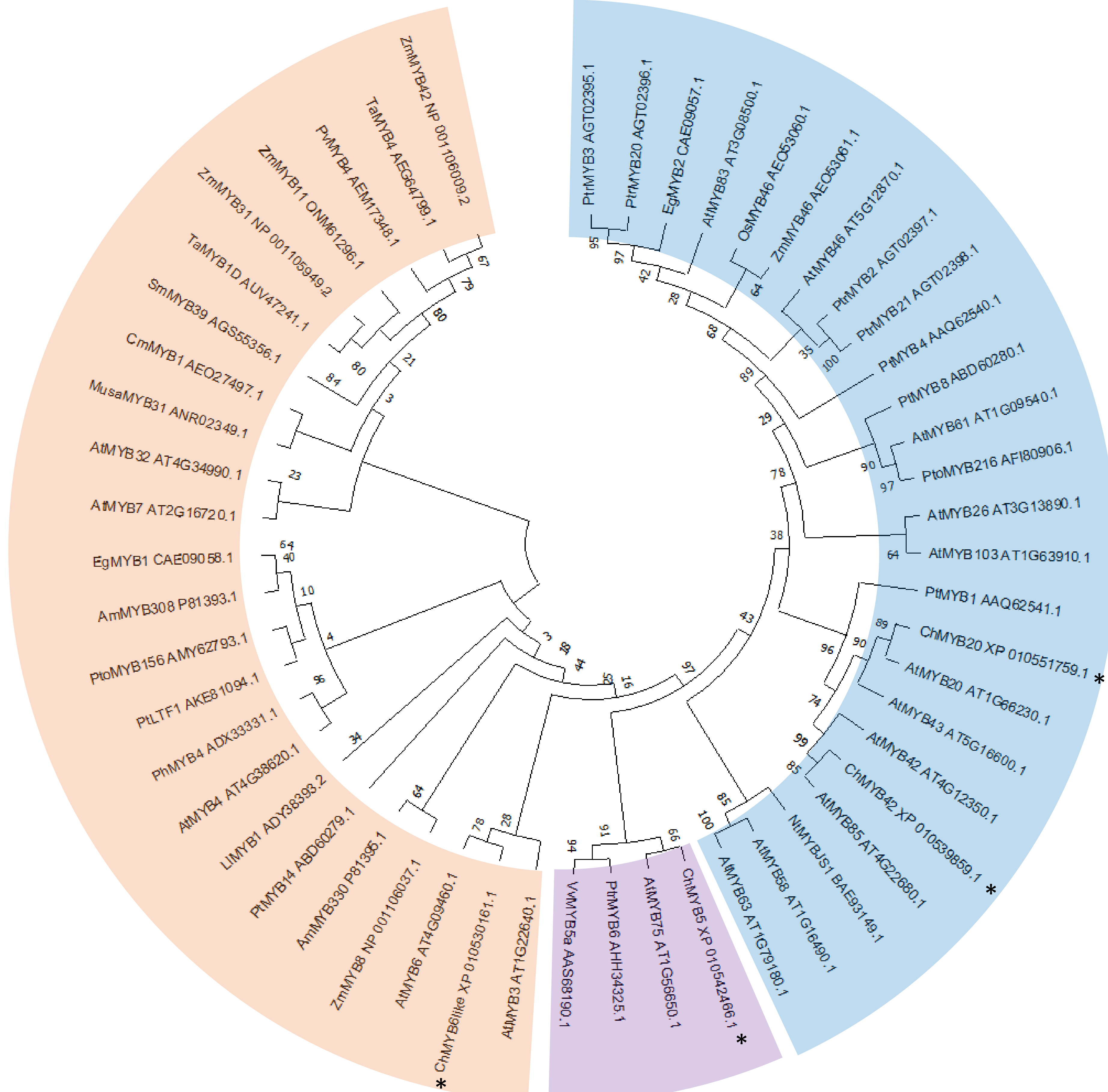

Figure S5. Phylogenetic analysis of ChMYB20, ChMYB6-like, ChMYB5 and ChMYB42 together with other MYBs known to regulate secondary wall biosynthesis or phenylpropanoid metabolism.

Protein sequences were obtained from GenBank. The phylogenetic tree was constructed using the Neighbor-joining method (Saitou and Nei, 1987) with 1000 bootstrap replicates by the software MEGA 11 (Molecular Evolutionary Genetics Analysis version 11, Tamura et al., 2021). Numbers indicate confidence percentages. Blue indicates the activator clade, orange indicates the repressor clade, purple indicates the third clade.

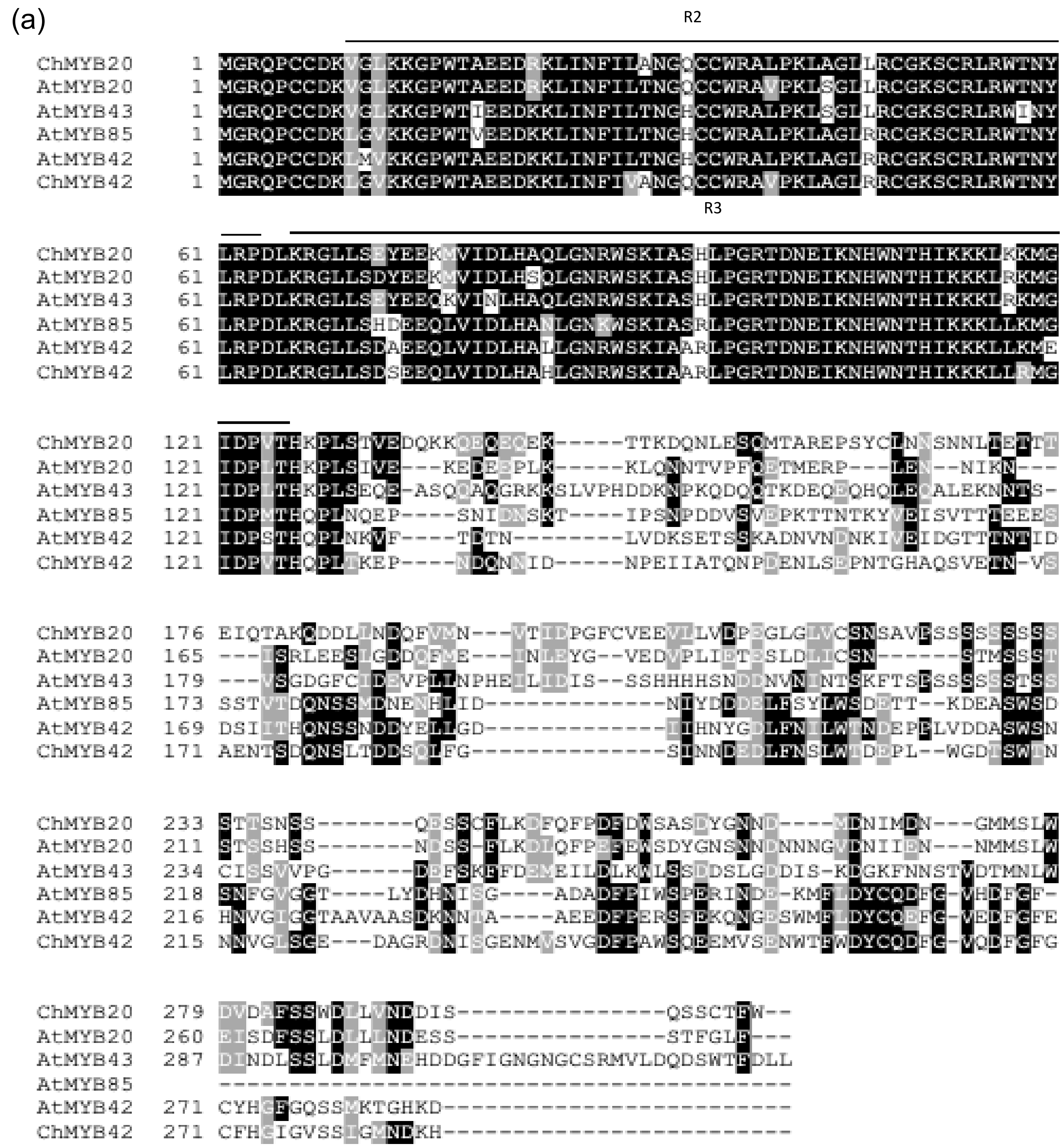

Figure S6. Multiple sequence alignments of ChMYB20 and ChMYB42 (a), ChMYB6-like (b) and ChMYB5 (c) with closely related MYBs.

(b)

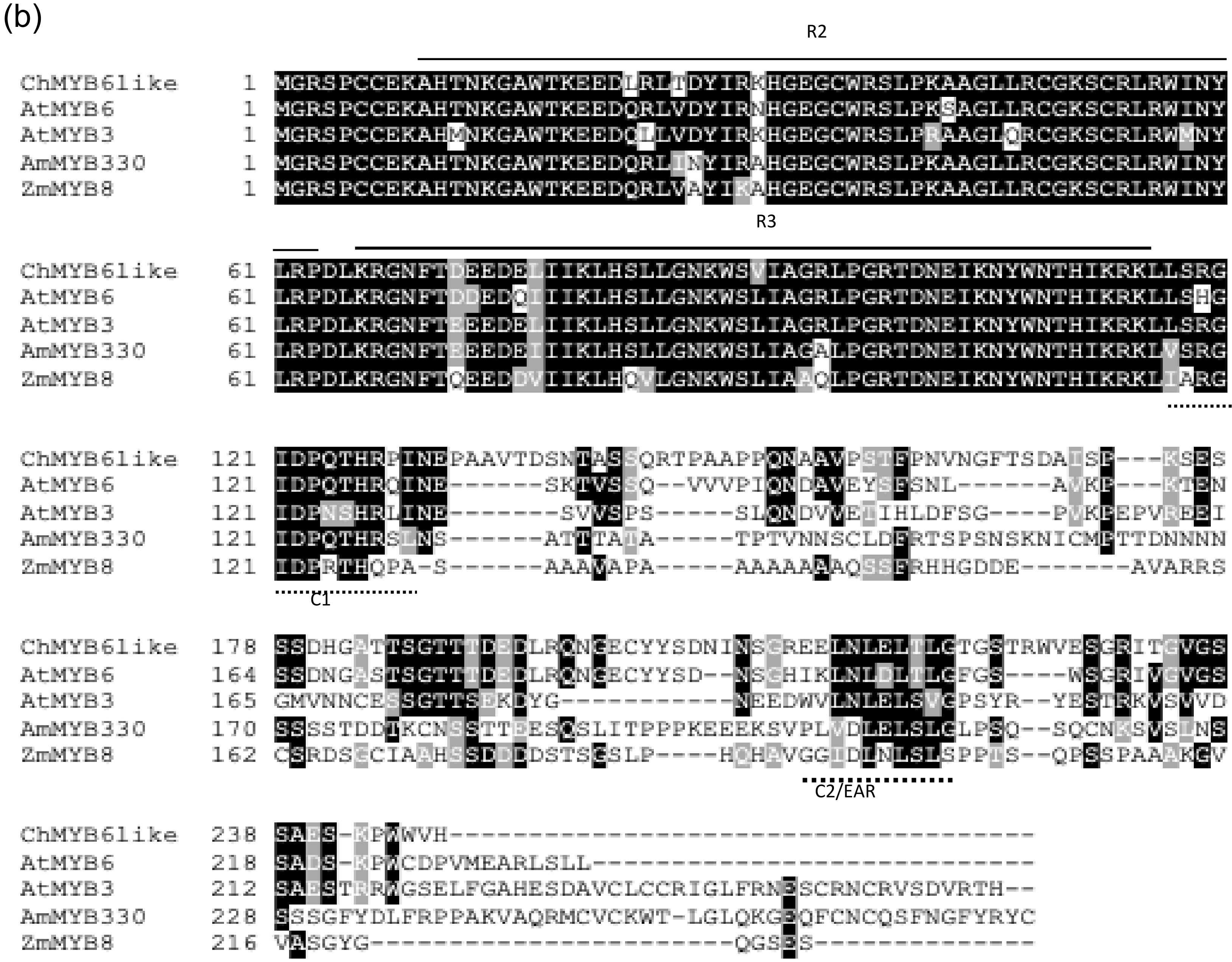
R2
ChMYB6like 1 MGRSPCCEKAHTNKGAWTKEEDLRLTDYIRKHGEGCWRSLPKAAGLLRCGKSCRLRWINY
AtMYB6 1 MGRSPCCEKAHTNKGAWTKEEDQRLVDYIRNHGEGCWRSLPKSAGLLRCGKSCRLRWINY
AtMYB3 1 MGRSPCCEKAHMNKGAWTKEEDQLLVDYIRKHGEGCWRSLPRAAGLQRCGKSCRLRWMNY
AmMYB330 1 MGRSPCCEKAHTNKGAWTKEEDQRLINYIRAHGEGCWRSLPKAAGLLRCGKSCRLRWINY
ZmMYB8 1 MGRSPCCEKAHTNKGAWTKEEDQRLVAYIKAHGEGCWRSLPKAAGLLRCGKSCRLRWINY
R3
ChMYB6like 61 LRPDLKRGNFTDEEDELIIKLHSLLGNKWSVIAGRLPGRTDNEIKNYWNTHIKRKLLSRG
AtMYB6 61 LRPDLKRGNFTDDEDQIIIKLHSLLGNKWSLIAGRLPGRTDNEIKNYWNTHIKRKLLSHG
AtMYB3 61 LRPDLKRGNFTEEEDELIIKLHSLLGNKWSLIAGRLPGRTDNEIKNYWNTHIKRKLLSRG
AmMYB330 61 LRPDLKRGNFTEEEDEIIIKLHSLLGNKWSLIAGALPGRTDNEIKNYWNTHIKRKLVSRG
ZmMYB8 61 LRPDLKRGNFTQEEDDVIIKLHQVLGNKWSLIAAQLPGRTDNEIKNYWNTHIKRKLIARG
ChMYB6like 121 IDPQTHRPINEPAAVTDSNTASSQRTPAAPPQNAAVPSTFPNVNGFTSDAISP---KSES
AtMYB6 121 IDPQTHRQINE------SKTVSSQ--VVVPIQNDAVEYSFSNL------AVKP---KTEN
AtMYB3 121 IDPNSHRLINE-------SVVSPS-----SLQNDVVETIHLDFSG----PVKPEPVREEI
AmMYB330 121 IDPQTHRSLNS------ATTTATA-----TPTVNNSCLDERTSPSNSKNICMPTTDNNNN
ZmMYB8 121 IDPRTHQPA-S------AAAVAPA-----AAAAAAAQSSERHHGDDE-------AVARRS
C1
ChMYB6like 178 SSDHGATTSGTTTDEDLRQNGECYYSDNINSCREELNLELTLGTGSTRWVESGRITGVGS
AtMYB6 164 SSDNGASTSGTTTDEDLRQNGECYYSD--NSCHIKLNLDLTLGFGS----WSGRIVGVGS
AtMYB3 165 GMVNNCESSGTTSEKDYG-----------NEEDWVLNLELSVGPSYR--YESTRKVSVVD
AmMYB330 170 SSSSTDDTKCNSSTTEESQSLITPPPKEEEKSVPLVDLELSLGLPSQ--SQCNKSVSLNS
ZmMYB8 162 CSRDSGCIAAHSSDDDDSTSGSLP----HQHAVGGIDLNLSLSPPTS--QPSSPAAAKGV
C2/EAR
ChMYB6like 238 SAES-KPWWVH-------------------------------------
AtMYB6 218 SADS-KPWCDPVMEARLSLL----------------------------
AtMYB3 212 SAESTRRWGSELFGAHESDAVCLCCRIGLFRNESCRNCRVSDVRTH--
AmMYB330 228 SSSGFYDLFRPPAKVAQRMCVCKWT-LGLQKGEQFCNCQSFNGFYRYC
ZmMYB8 216 VASGYG----------------------QGSES---------------

(c)

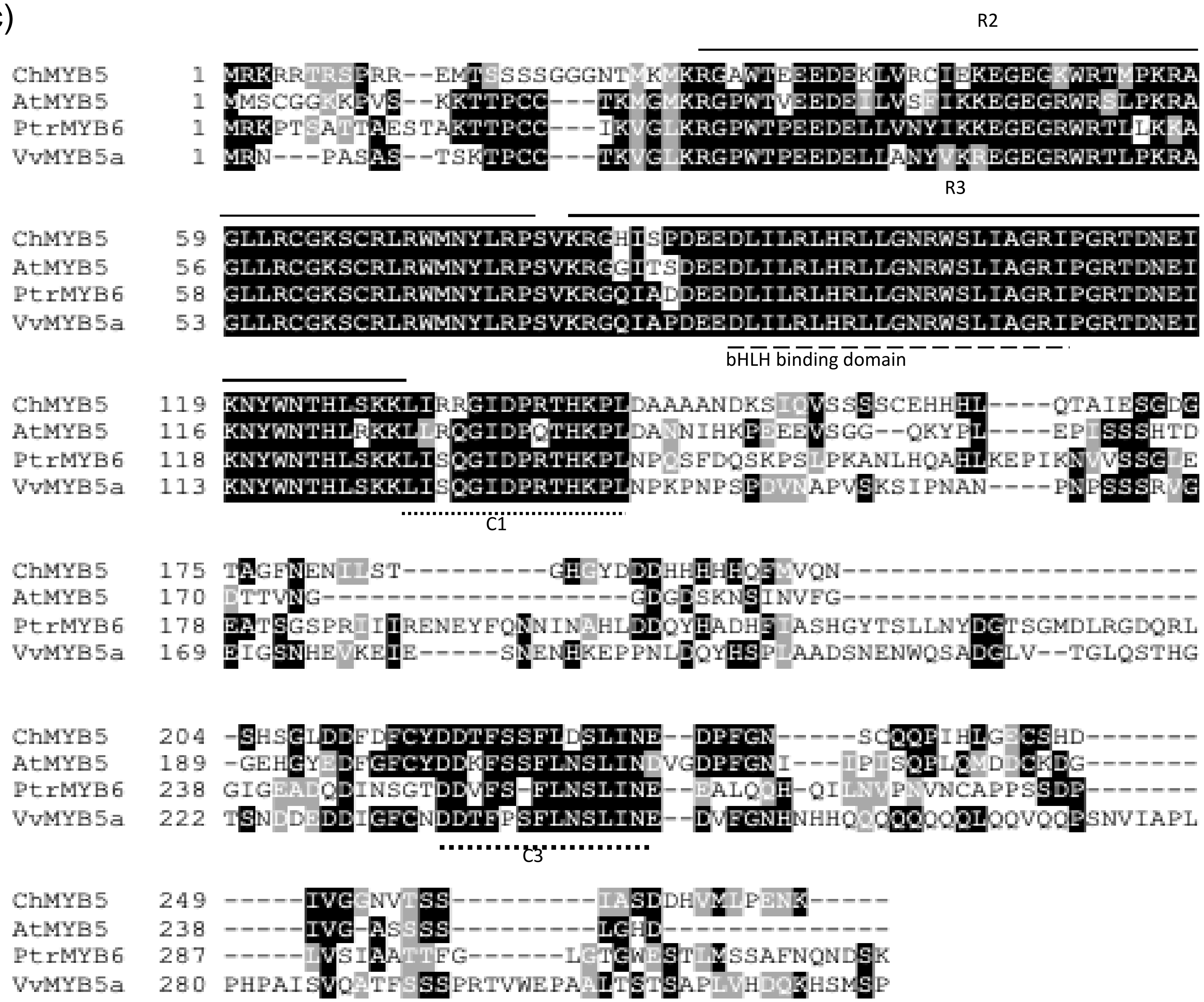

R2
ChMYB5 1 MRKRRTRSPRR--EMTSSSSGGGNTVKMKRGAWTEEEDEKLVRCIEKEGEGKWRTMPKRA
AtMYB5 1 MMSCGGKKPVS--KKTTPCC---TKMGMKRGPWTVEEDEILVSFIKKEGEGRWRSLPKRA
PtrMYB6 1 MRKPTSATTAESTAKTTPCC---IKVGLKRGPWTPEEDELLVNYIKKEGEGRWRTLLKKA
VvMYB5a 1 MRN---PASAS--TSKTPCC---TKVGLKRGPWTPEEDELLANYVKREGEGRWRTLPKRA
R3
ChMYB5 59 GLLRCGKSCRLRWMNYLRPSVKRGHISPDEEDLILRLHRLLGNRWSLIAGRIPGRTDNEI
AtMYB5 56 GLLRCGKSCRLRWMNYLRPSVKRGGITSDEEDLILRLHRLLGNRWSLIAGRIPGRTDNEI
PtrMYB6 58 GLLRCGKSCRLRWMNYLRPSVKRGQIADDEEDLILRLHRLLGNRWSLIAGRIPGRTDNEI
VvMYB5a 53 GLLRCGKSCRLRWMNYLRPSVKRGQIAPDEEDLILRLHRLLGNRWSLIAGRIPGRTDNEI
bHLH binding domain
ChMYB5 119 KNYWNTHLSKKLIRRGIDPRTHKPLDAAAANDKSIQVSSSSCEHHHL----QTAIESGDG
AtMYB5 116 KNYWNTHLRKKLLRQGIDPQTHKPLDANNIHKPEEEVSGG--QKYPL----EPISSSHTD
PtrMYB6 118 KNYWNTHLSKKLISQGIDPRTHKPLNPQSFDQSKPSLPKANLHQAHLKEPIKNVVSSGLE
VvMYB5a 113 KNYWNTHLSKKLISQGIDPRTHKPLNPKPNPSPDVNAPVSKSIPNAN----PNPSSSRVG
C1
ChMYB5 175 TAGFNENIISST---------GHGYDDDHHHHQENVQN---------------------
AtMYB5 170 DTTVNG------------------GDGDSKNSINVFG---------------------
PtrMYB6 178 EATSGSPRIIIRENEYFQNNINAHLDDQYHADHEIASHGYTSLLNYDGTSGMDLRGDQRL
VvMYB5a 169 EIGSNHEVKEIE-----SNENHKEPPNLDQYHSPLAADSNENWQSADGLV--TGLQSTHG
ChMYB5 204 -SHSGLDDFDFCYDDTFSSFLDSLINE--DPFGN-----SCQQPIHLGECSHD-------
AtMYB5 189 -GEHGYEDFGFCYDDKFSSFLNSLINDVGDPFGNI---IPIISQELQMDDCKDG-------
PtrMYB6 238 GIGEADQDINSGTDDVFS-FLNSLINE--EALQQH-QIILNVPNVNCAPPSSDP-------
VvMYB5a 222 TSNDDEDDIGFCNDDTFPSFLNSLINE--DVFGNHNHHQQQQQQQQLQQVQQPSNVIAPL
C3
ChMYB5 249 -----IVGGNVTSS---------IASDDHVMIPENK------
AtMYB5 238 -----IVG-ASSSS---------LGHD-------------
PtrMYB6 287 -----IVSIAATTFG------LGTGWESTLMSSAFNQNDSK
VvMYB5a 280 PHPAISVQATFSSSPRTVWEPAALTSTSAPLVHDQKHSMSP

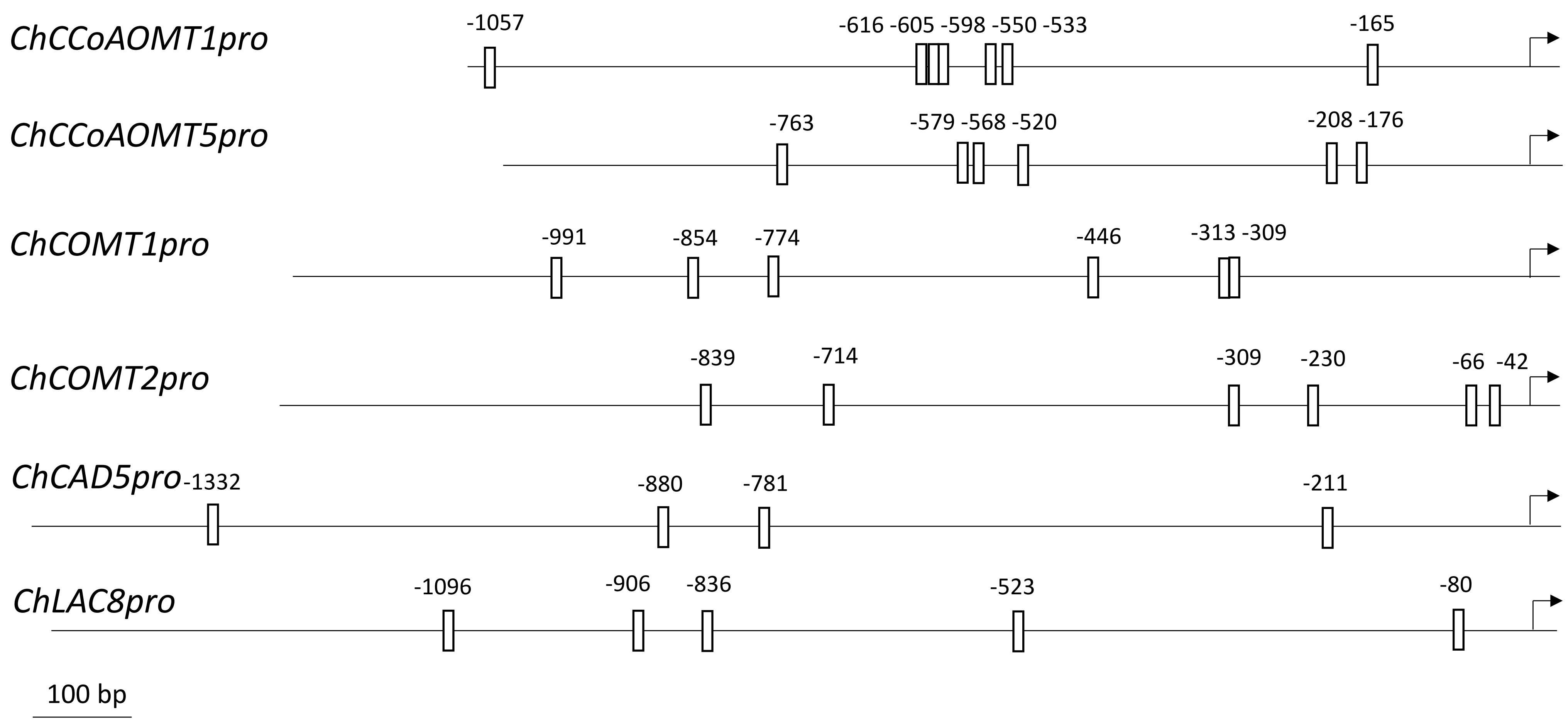

Figure S7. cis-Element analysis of ChCCoAOMTs, ChCOMTs, ChCAD5 and ChLAC8 promoters. A square represents AC elements with the consensus sequences ACCTNNC (Mellway et al., 2009) or secondary wall MYB-responsive elements (SMRE) with the consensus sequences ACC(A/T)A(A/C)(T/C) (Zhong and Ye, 2012) in promoter regions, and numbers indicate the position of these motifs relative to the translational start (marked by an arrow).

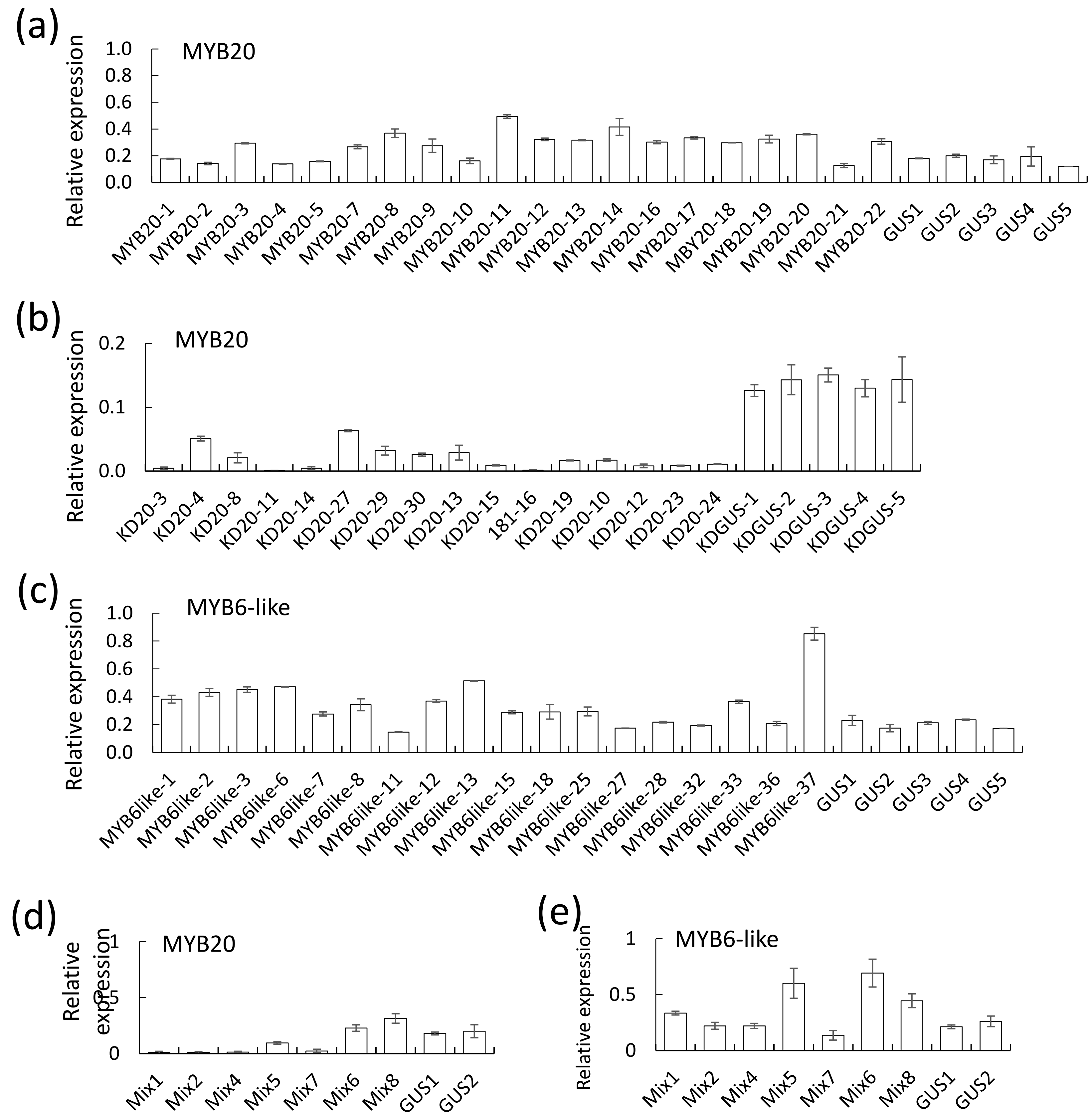

Figure S8. Transgene transcript levels in Cleome hairy roots overexpressing ChMYB20, ChMYB6-like and knockdown of ChMYB20.

Transcript levels of ChMYB20 (a, b, d) or ChMYB-like (c, e) in transgenic Cleome hairy roots overexpressing ChMYB20 (a), knockdown of ChMYB20 (b), overexpressing ChMYB6-like (c), and with the combination of knockdown of ChMYB20 and overexpressing ChMYB6-like (d,e). Transcript levels was determined by qRT-PCR expressed relative to ubiquitin-conjugating enzyme E2 11 (*UBC*). Data are means of three biological replicates.

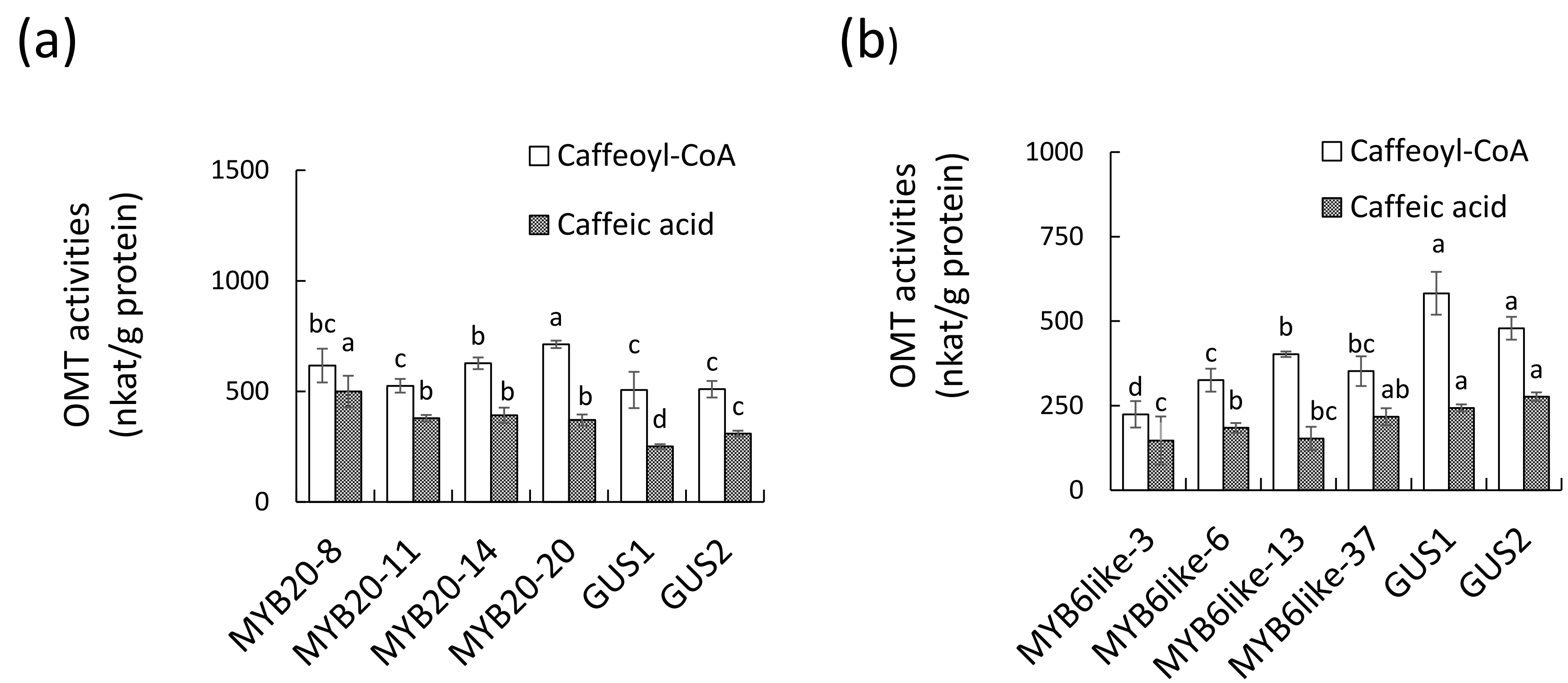

Figure S9. Effect of expression of ChMYB20 or ChMYB6-*like* on 3-*O*-methyltransferase enzymatic activity in Cleome hairy roots.

Extractable 3-*O*-methyltransferase enzymatic activity on caffeoyl-CoA (for CCoAOMT) and caffeic acid (for COMT) in Cleome hairy root cultures overexpressing ChMYB20 (a) or ChMYB6-like (b).

Four independent lines overexpressing ChMYB20 or ChMYB6-like were analyzed and compared with two lines expressing GUS. Data are means of three biological replicates. Letters indicate significant differences at the $P \leq 0.05$ level by one-way ANOVA (Duncan) with SPSS Statistics.

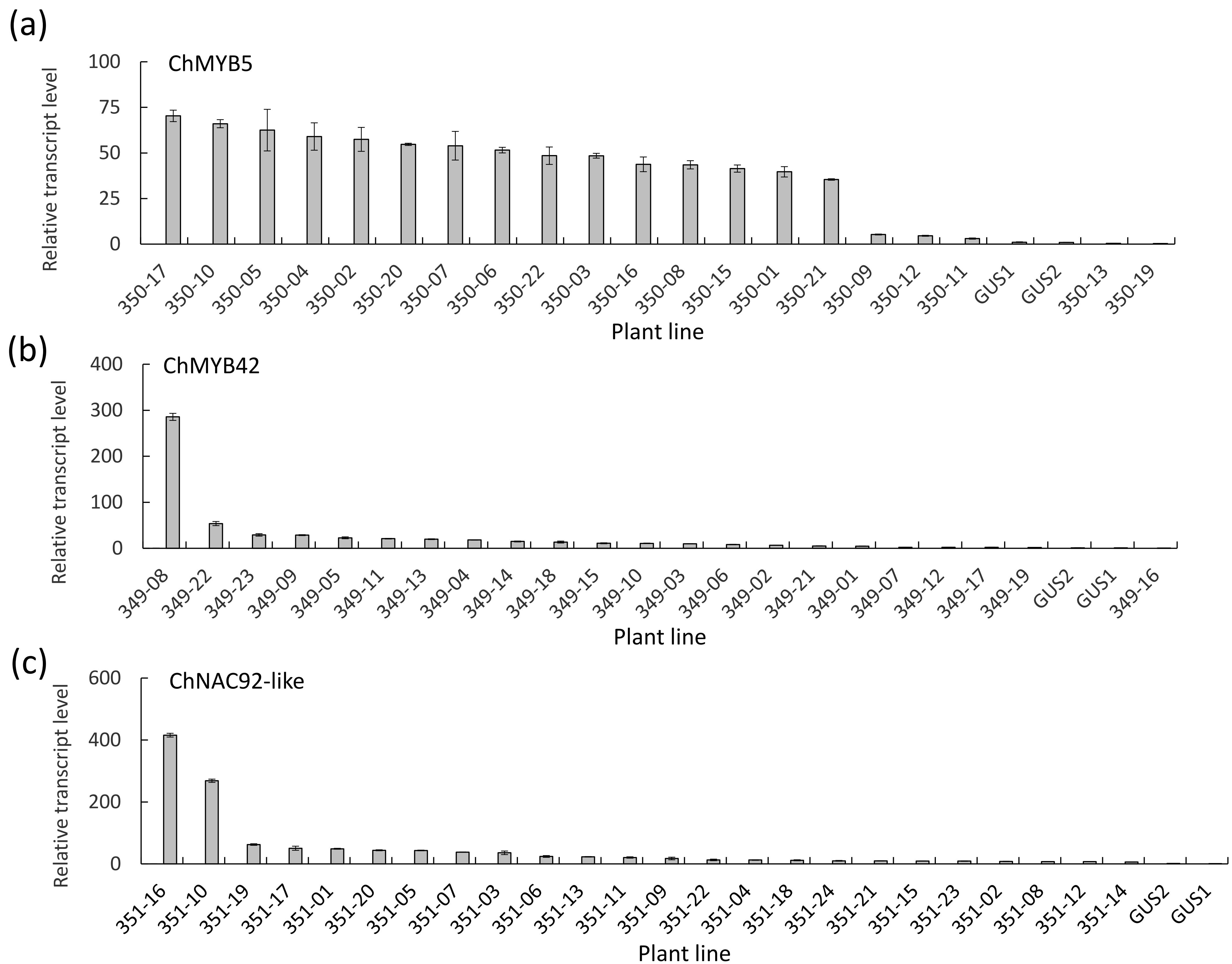

Figure S10. Transcript levels of transcription factors in Cleome hairy roots overexpressing ChMYB5 (a), ChMYB42 (b) or ChNAC92-like (c), as determined by qRT-PCR. The Cleome ubiquitin-conjugating enzyme E2 11-like gene was used as an internal standard to normalize the amount of cDNA template. The average transcript levels in the GUS lines was set as 1. The top eight lines with the highest transcript levels were selected for lignin assays by thioacidolysis.

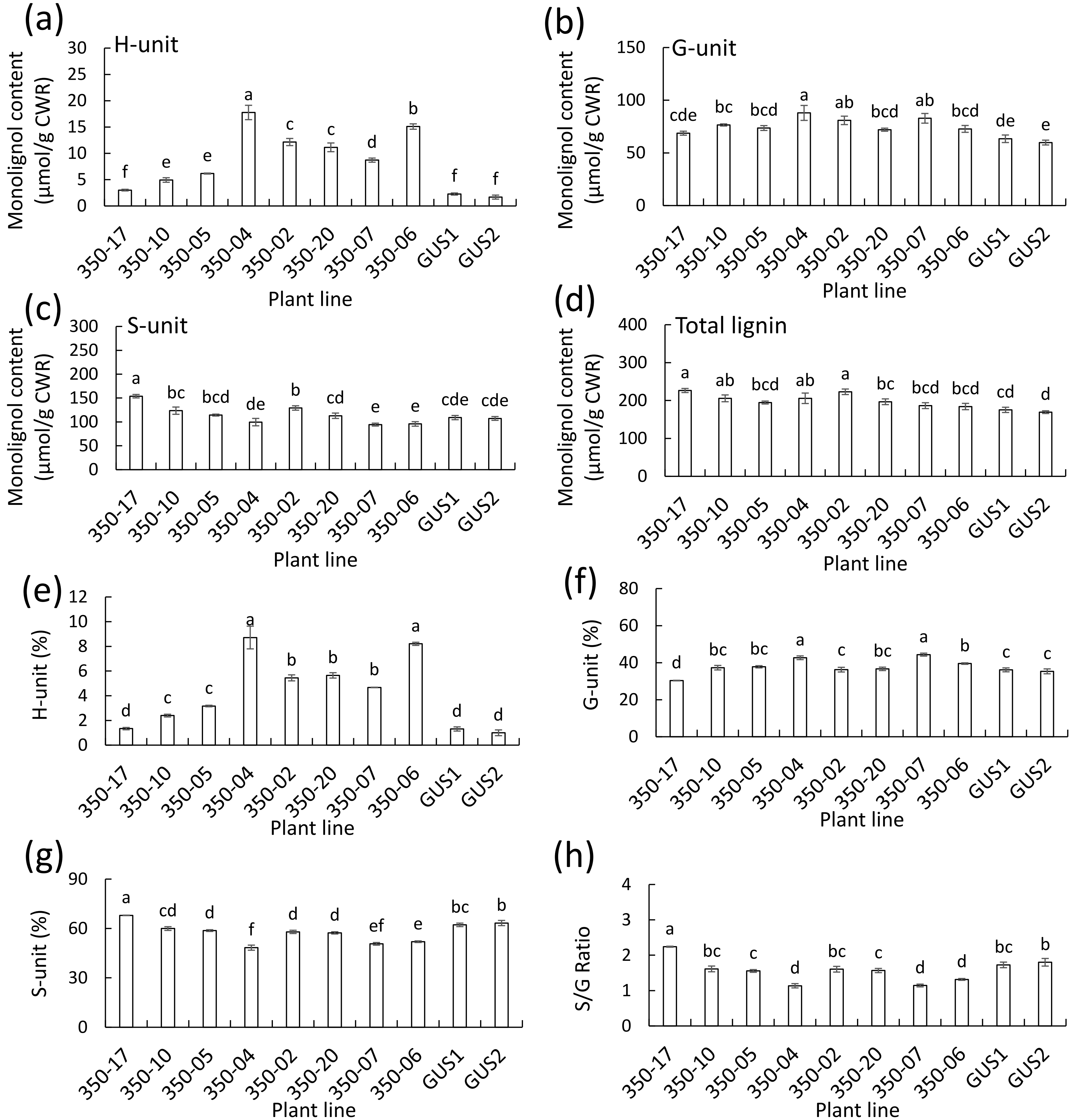

Figure S11. Lignin content and composition in Cleome hairy roots overexpressing ChMYB5. (**a-d**) Total monomer units and lignin amount as determined by thioacidolysis. CWR, cell wall residue. (**e-g**) Percentage of H, G and S units in the above lignin samples. (**h**) S/G ratio.

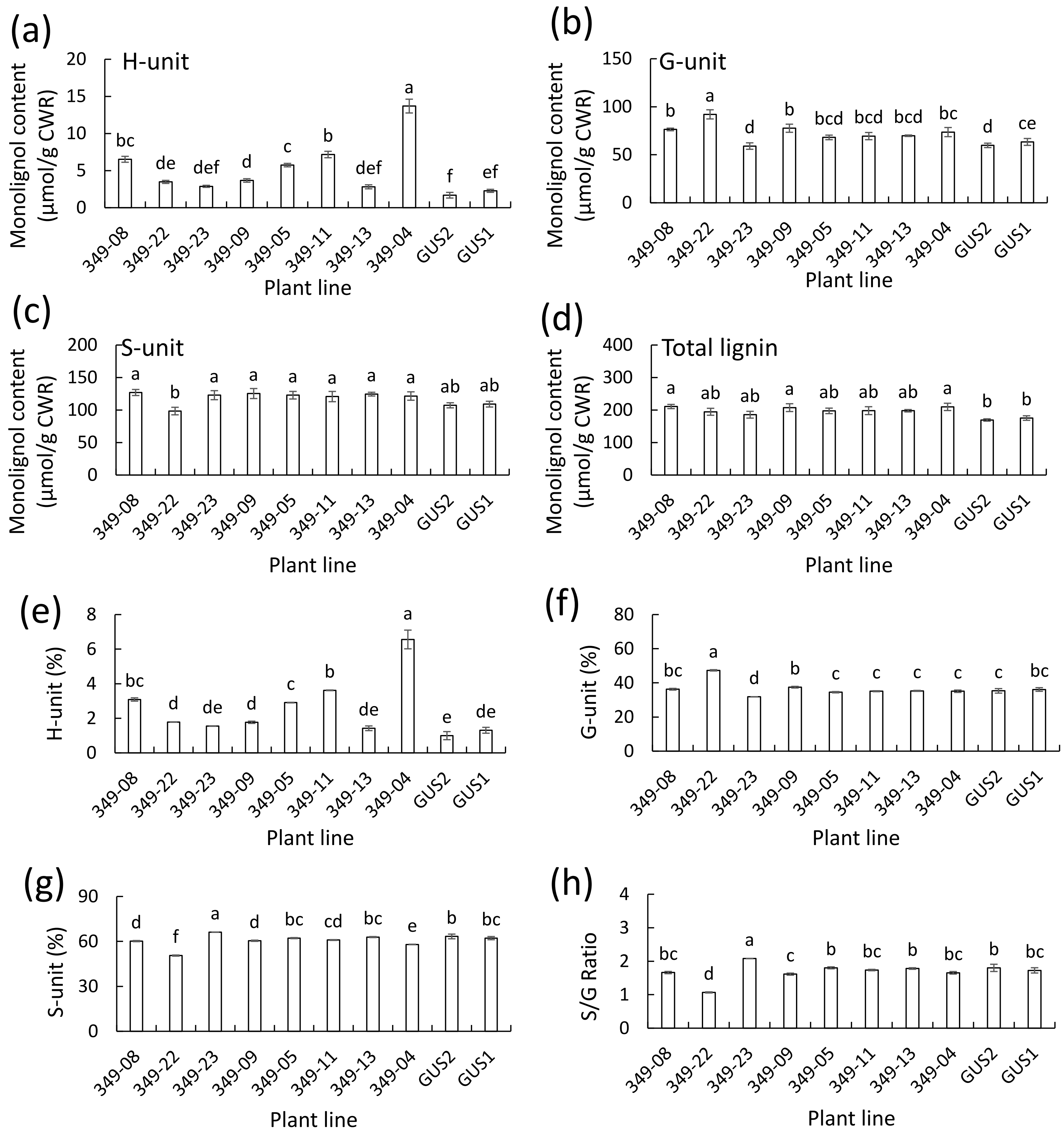

Figure S12. Lignin content and composition in Cleome hairy roots overexpressing ChMYB42.
(**a-d**) Total monomer units and lignin amount as determined by thioacidolysis. CWR, cell wall residue.
(**e-g**) Percentage of H, G and S units in the above lignin samples.
(**h**) S/G Ratio.

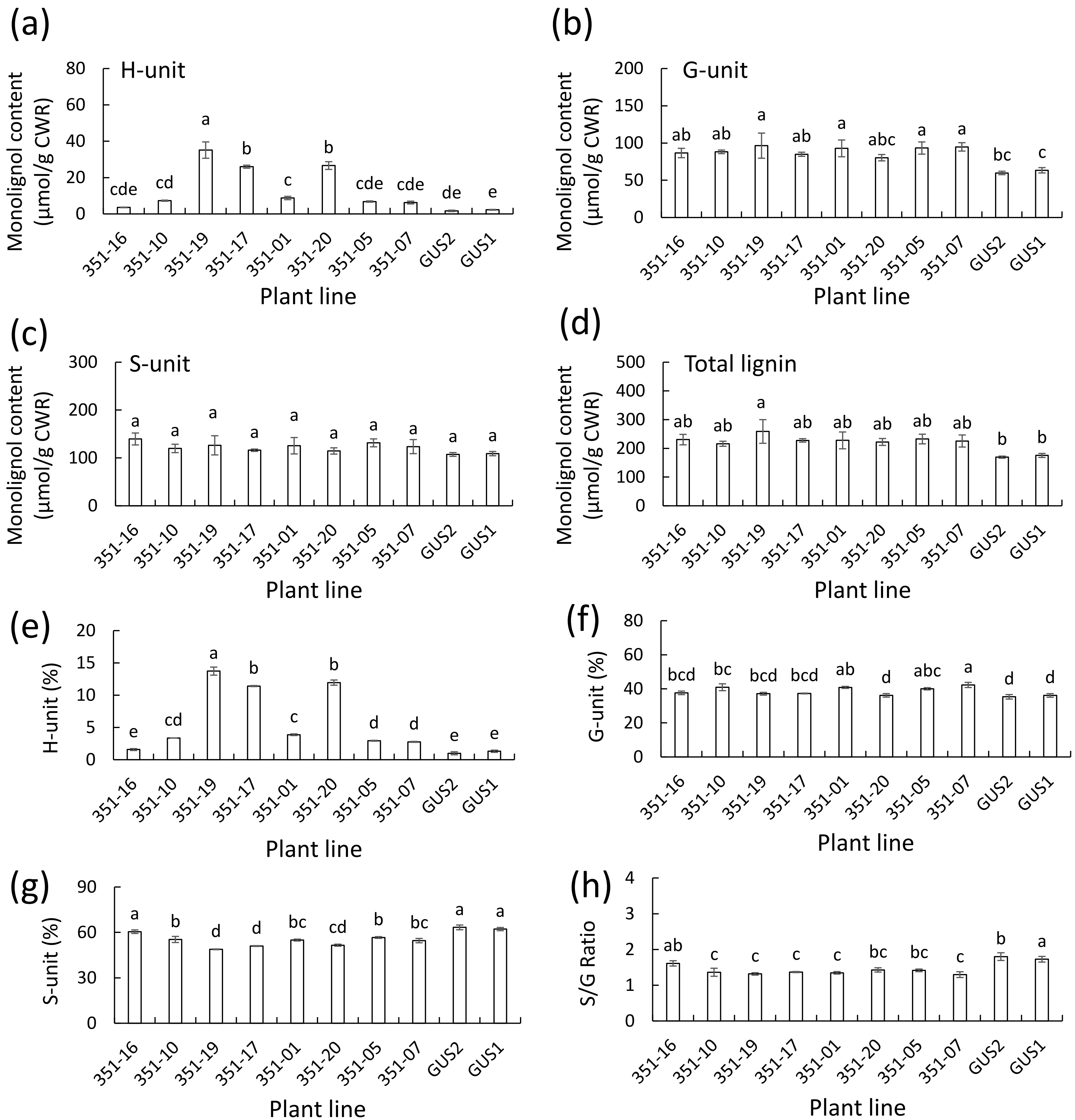

Figure S13. Lignin content and composition in Cleome hairy roots overexpressing ChNAC92-like.
(**a-d**) Total monomer units and lignin amount as determined by thioacidolysis. CWR, cell wall residue.
(**e-g**) Percentage of H, G and S units in the above lignin samples.
(**h**) S/G ratio.

Table S1. Fold-change of lignin-related structural and regulatory gene transcript levels in Cleome hairy roots overexpressing ChNAC92-like, ChMYB5 or ChMYB42

Two to four TF overexpressing lines were used to check transcript levels of potential target genes. The Cleome ubiquitin-conjugating enzyme E2 11-like gene was used as an internal standard to normalize the amount of cDNA template. The average transcript levels in the GUS lines were used to calculate fold change. Data are means derived from three biological replicates. Genes with similar expression pattern were grouped together. The $\log_2$ fold change is visualized as a Heatmap in Figure 8

	NAC92-like				*MYB5*		*MYB42*	
	351-19	351-17	351-10	351-01	350-4	350-6	349-08	349-04
COMT1	2.4	5.1	2.8	4.8	0.8	0.5	0.2	0.6
COMT2	1.3	1.3	1.3	1.9	1.4	1.2	0.4	0.7
CCoAOMT1	8.1	12.4	4.2	8.2	2.6	2.6	0.6	0.6
CCoAOMT5	4.7	3.6	3.5	5.8	1.5	1.3	1.0	0.9
MS	23.3	9.5	9.0	16.3	2.3	1.0	1.2	1.1
CCR1	5.1	7.9	7.8	8.1	1.6	2.0	1.3	1.1
CAD4	13.5	10.7	11.4	16.8	4.9	4.1	1.4	1.4
LAC4	15.8	7.2	11.7	35.1	29.8	13.4	1.9	4.9
MYB20	2.4	1.8	2.1	1.5	1.5	2.4	1.4	1.9
MYB6-like	7.7	5.9	7.6	10.0	11.1	10.5	2.2	2.5
ERF105	9.9	18.5	5.9	13.8	12.3	10.3	1.8	2.3
NAC92-like	59.6	45.2	378.1	48.9	6.8	3.7	2.7	1.8
MYB5	2.1	1.4	2.5	1.5	68.4	74.7	3.5	4.6
ERF015	1.2	0.9	0.8	1.0	21.4	15.0	1.1	1.3
MYB42	2.9	2.6	4.1	2.3	31.0	32.8	216.1	35.7
SUP	4.4	2.2	4.1	2.8	3.5	8.0	151.8	76.8
CAD5	4.0	2.1	5.8	4.2	67.0	87.8	8.6	7.9
LAC8	1.8	1.0	4.0	1.4	81.8	91.5	3.6	6.4
PLT3	5.8	3.7	2.2	3.0	29.2	22.8	3.1	4.1
SUC1	0.9	0.8	1.1	0.8	36.9	33.4	3.2	3.9

Table S2. Primers used in the present work.

Oligo names	Sequence (5' -> 3')	Purpose
NAC43 F	CACCATGTCGGAAGCTATGAACATATCCGTG	Cloning ORF
NAC43 R	TTATCCACTACCGTTCGACACGTG	Cloning ORF
NAC73 F	CACCATGACATGGTGCAACGACTGCAG	Cloning ORF
NAC73R	TCAAGGGATAAAAGAAGGAGCTTCATGAAC	Cloning ORF
MBY20 F	CACCATGGGGAGACAGCCATGTTGTGAC	Cloning ORF
MYB20 R	TTACCAAAATGTGCAAGAACTCTGAGAAATATC	Cloning ORF
MYB69 F	CACCATGGAAATCTCAAGAACGAGCAACAG	Cloning ORF
MYB69 R	CTAAGAAGCTAATCCAACCCCAAG	Cloning ORF
MYB86 F	CACCATGGGGCGACATTCTTGTTGTTACAAG	Cloning ORF
MYB86 R	CTAAAGGGTCTGACCAAATGGCAC	Cloning ORF
MYB118 F	CACCATGGATTTTGATGTTGTTTTCAGAACGC	Cloning ORF
MYB118 R	CTAAGCACTTCCACCACCGG	Cloning ORF
RAP2-3 F	CACCATGTGTGGCGGTGCAATAATTTCCG	Cloning ORF
RAP2-3 R	TTAACACAAGTACCTGCCATGGCC	Cloning ORF
RAP2-3-like F	CACCATGTGTGGTGGTGCGATAATTTCCG	Cloning ORF
RAP2-3-like R	ATCACACGGACGAACGATGCCC	Cloning ORF
TT16 F	CACCATGGGGAGAGGAAAGATAGAGATAAAG	Cloning ORF
TT16 R	TCAATCTTGAAGATTAGGCTGAGCGAG	Cloning ORF
SEP3 F	CACCATGGGAAGAGGTCGTGTTGAGTTG	Cloning ORF
SEP3 R	TCAATTGTTGTCATAAGGTAACCAACC	Cloning ORF
MYB6-like F	CACCATGGGAAGGTCGCCTTGCTGC	Cloning ORF
MYB6-like R	TCAGTGGACCCACCAAGGCTTC	Cloning ORF
WRKY57 F	CACCATGGACGATCCCGGCGAGAAC	Cloning ORF
WRKY57 R	TCATCGGTTACGCATGGAGGGAG	Cloning ORF
NAC40 F	CACCATGGGAATGTACGACGAGTGTACTTC	Cloning ORF
NAC40 R	TTAGCCGGAAAAAGCAGCCCAAAC	Cloning ORF
NAC92-like F	CACCATGGCGGTGGTAGAAGAAGGCGTAG	Cloning ORF
NAC92-like R	TTAGTAGTCCCATAAGATTCCCAAGTCTCC	Cloning ORF
MYB5 F	CACCATGAGGAAGAGGAGGACGAGGTC	Cloning ORF
MYB5 R	TCACTTGTTTTCAGGCAGCATAACATGATC	Cloning ORF
MYB42 F	CACCATGGGGAGGCAACCGTGTTGTG	Cloning ORF
MYB42 R	CTAATGCTTGTCATTCATCCCAATGGAAC	Cloning ORF
ERF015 F	CACCATGGATATGATGCCGCCCTCTC	Cloning ORF
ERF015 R	TCACAGTAGATCGTTAATCCAGTCTATG	Cloning ORF
ERF105 F	CACCATGGCGAATGGCCGAGAACAATC	Cloning ORF
ERF105 R	TCAGGTAACCACAATTTGGGAGTACC	Cloning ORF
SHINE2 F	CACCATGGTACAGAATGCAAAGAAGTTCCGAG	Cloning ORF
SHINE2 R	TTAATTAGACTCAAATGGAAGCAGATTAGG	Cloning ORF

AGL1 F	CACCATGCAGCAGTTAAGTGTTGGAGATAAAGTC	Cloning ORF
AGL1 R	TTAGACGAGCTGGAGAGGGGG	Cloning ORF
AGL11 F	CACCATGTCTTCGTGGATCAGTTTAACTGCC	Cloning ORF
AGL11 R	TCAGCCGAGATGGAGGGTTCTC	Cloning ORF
bZIP43 F	CACCATGGAACCTAATGACCGTCCCTC	Cloning ORF
bZIP43 R	TCAAGTTTTGTGTGGTCGGAGGTTG	Cloning ORF
bZIP44 F	CACCATGAATAAATCTGAAATGGGTTCGTCTTCGAC	Cloning ORF
bZIP44 R	TCATGGACAGTGGAAGATATCAGCAGC	Cloning ORF
SUP F	CACCATGGAGAGAAACGACGTAGATTTGACG	Cloning ORF
SUP R	CTATGCGATGCCCAAGCGGAG	Cloning ORF
ZAT12 F	CACCATGGAGGCGAAGTGCTTGATGTTATTG	Cloning ORF
ZAT12 R	TCAATGAACTGTTCTTCCGAGCTCC	Cloning ORF
CCoAOMT1 pro1.4k F	CACCGACCCTCACCTGTTATCATTCG	Cloning promoter
CCoAOMT1 pro1k F	CACCGGGGAGAAACATCACCAAATATTC	Cloning promoter
CCoAOMT1 +144 R	CTGATACAGATCATCGCTCTGTAA	Cloning promoter
CCoAOMT1 pro -45 R	GGATGAACATGGAATATAAAGGAGGAAG	Cloning promoter
CCoAOMT5 pro1.6k F	CACCTCGCTAACGAATTTTAAACTTGAAAC	Cloning promoter
CCoAOMT5 pro1k F	CACCCAATCCCCTATAATTGAAATCAAAC	Cloning promoter
CCoAOMT5 +123 R	AGCATCGCTCTGCAGAAGACT	Cloning promoter
CCoAOMT5 pro -29 R	CTCTGTTAATATCTCTGCTTTAATGGAG	Cloning promoter
COMT1 pro1.5k F	CACCCTCTTTAACGTTGTATTCTG	Cloning promoter
COMT1 pro1.1k F	CACCGTCGAAATTGAATTATCATATAGAGTG	Cloning promoter
COMT1 +55 R	CTTCGTCGTCGGAGACTTGGTT	Cloning promoter
COMT1 pro -44 R	CAGATTTGTCTTAGTGGGGATTGG	Cloning promoter
COMT2 pro1.5k F	CACCGTTAGTGATGAACTTAACAACTTTCT	Cloning promoter
COMT2 pro1.3k F	CACCGTGTCCACATTTGTTCTAGC	Cloning promoter
COMT2 +250 R	CCAGGATCCGGAGAATGCGA	Cloning promoter
COMT2 pro -23 R	GTGTATTTTTCAGGTTTGGTGAGTGAG	Cloning promoter
CAD5 pro1.5k F	CACCTGGAAGGAGAGAAAGAGGC	Cloning promoter
CAD5 pro1.1k F	CACCACTATCAGAATAGTCAAACGTAGAGCATAGC	Cloning promoter
CAD5 +85 R	GAGTGTATGTGTAAGGCGAGAGGAACC	Cloning promoter
CAD5 pro -25 R	GTTTTGATATTTTAGAGGCACTACATGTGTG	Cloning promoter
LAC8 pro1.5k F	CACCTCTAGACCCGTGACAGTAGCG	Cloning promoter
LAC8 pro1k F	CACCTTAGGTATATGAAAGACACTTGCGTCCG	Cloning promoter
LAC8 +96 R	GAAAGTGTGTTCGACGACAGATGC	Cloning promoter
LAC8 pro -4 R	GGAGACGATGCCTTGGAGGAAG	Cloning promoter
MYB6-like pro2k F	CACCGCTCTTTGAAGACTACCACATAAACTATGC	Cloning promoter
MYB6-like pro1.5k F	CACCGTGGTCTTGTCTTCAACCTCGGAC	Cloning promoter
MYB6 +110 R	CAACAGCCTTCGCCGTGTTTACG	Cloning promoter
MYB6 pro -1 R	GATGATGCCTGTCTATGTATGGATGTC	Cloning promoter
MYB5 pro2k F	CACCGCACACCTCCTTGGTTGTCAAAATG	Cloning promoter

MYB5 pro1.5k F	CACCTCACTCCAGAATAATATGAAGGTGTGTC	Cloning promoter
MYB5 +123 R	CCTCACCAGCTTCTCGTCCTC	Cloning promoter
MYB5 pro -26 R	CTCTCTCTTCCTCTCTTTCTCTCTC	Cloning promoter
MYB42 pro2k F	CACCGAAAGTTGCAGAATAAAAAGTAGTGGGGG	Cloning promoter
MYB42 pro1.5k F	CACCTTGTGGGTAAAATCGCACAAGTCAAAAG	Cloning promoter
MYB42 +98 R	CCGTTCGCCACTATGAAGTTTATGAGC	Cloning promoter
MYB42 pro -28 R	GATACCTTATCACAGAGAGAGAGAGAG	Cloning promoter
MYB20 RNAi F	CACCGCTATTGTCTGAACAACAGCAACAACTTG	RNAi
MYB20 RNAi R	CGAAGTCGTCGAAGATGAAGAAGAAG	RNAi
MYB20 qPCR F	GCGAAGCAGGACGATTTACTCAAC	qPCR
MYB20 qPCR R	GAGGAAGAAGAGGGAACTGCAG	qPCR
MYB6-like qPCR F	CAACACGGCGTCGTCTCAGAG	qPCR
MYB6-like qPCR R	GGCTCCGTGGTCTGAGGAAG	qPCR
NAC92-like qPCR F	CCA CAC ACC GAT CTA CAA CAA CCC	qPCR
NAC92-like qPCR R	CGTTGCGGTCGCGTTCATGTC	qPCR
MYB5 qPCR F	GAA CTG CAG GCT TCA ACG AGA ATA TTC	qPCR
MYB5 qPCR R	GGGTCTTCATTGATGAGGGAATCCAG	qPCR
MYB42 qPCR F	CGT TGG ACT TAG TGG AGA AGA CGC	qPCR
MYB42 qPCR R	CCAAAACGTCCAATTTTCGCTGACC	qPCR
COMT1 qPCR F	GCATTCTTCCCGTGGCTCCTGAT	qPCR
COMT1 qPCR R	GCAGACGACTTGGAAGCCATGG	qPCR
COMT2 qPCR F	ATCATGTTGGCTCATAACCCCGGA	qPCR
COMT2 qPCR R	TTGCAGACAACCCGAAAACCCTGA	qPCR
CCoAOMT1 qPCR F	CGGTGGAAGCAACGAAGAAGGAG	qPCR
CCoAOMT1 qPCR R	CTGATACAGATCATCGCTCTGTAAGAG	qPCR
CCoAOMT5 qPCR F	CGACGAATGCGGAAACGACGAC	qPCR
CCoAOMT5 qPCR R	CAGAAGACTCTTGTGTCCGACTTCT	qPCR
MS1 qPCR F	GTTGGGTCCTGATGTGAAATTCTCTTATG	qPCR
MS1 qPCR R	CCTTGTAGATGGGAAGAATTTTCTCGAC	qPCR
CCR1 qPCR F	GACCGCCATTGCAGCCGG	qPCR
CCR1 qPCR R	GAGGGCGACTCGTAGACCAAAAC	qPCR
CAD4 qPCR F	GCCGACCATGCAATTCAGATCTC	qPCR
CAD4 qPCR R	GAACTTCTGATGGACGATCATAGACTC	qPCR
LAC4 qPCR F	CAAGAGCTGTCCCTCGTCCATG	qPCR
LAC4 qPCR R	GGCGATGAGTACGGTGTCTGTC	qPCR
ERF105 qPCR F	AACAATCAGCCCTGGAGCTCATAAG	qPCR
ERF105 qPCR R	CATCATCATCATCACTAGGCATCGG	qPCR
ERF015 qPCR F	CCACTGAATCTGTGAGGATCCC	qPCR
ERF015 qPCR R	TCACAGTAGATCGTTAATCCAGTCTATG	qPCR
SUP qPCR F	GACTCACCGTGGTGCCTTGG	qPCR
SUP qPCR R	GTCTCTCCTGTGAACATTCATGTGC	qPCR

CAD5 qPCR F	ATCCGACAAGAAGAGACGAGAAGC	qPCR
CAD5 qPCR R	GATGTAGTCGAGAGAATCCGCTAATC	qPCR
LAC8 qPCR F	GATAACACAATATCCGATCCGACCG	qPCR
LAC8 qPCR R	GAGAATGATGAGAGCTTCTTCATAGGG	qPCR
PLT3 qPCR F	GTTTTGATGGCCGGAAGGTG	qPCR
PLT3 qPCR R	CCGTAGTTCGAGACGTAGCC	qPCR
SUC1 qPCR F	CCGTTCGATGTGGCTCAAAC	qPCR
SUC1 qPCR R	TGAGTGAGGCCCATTTGTGG	qPCR
UBC qPCR F	GGTTGGATCGGCTGCAATACATAATC	qPCR
UBC qPCR R	CTTGAAGCCGCAGAGTCACGAG	qPCR
